# Anion Effects on the Supramolecular Self-Assembly of Cationic Phenylalanine Derivatives

**DOI:** 10.1021/acs.langmuir.2c01394

**Published:** 2022-12-06

**Authors:** Brittany L. Abraham, Pamela Agredo, Samantha G. Mensah, Bradley L. Nilsson

**Affiliations:** † Department of Chemistry, University of Rochester, Rochester, New York 14627-0216, United States; ‡ Materials Science Program, University of Rochester, Rochester, New York 14627-0166, United States

## Abstract

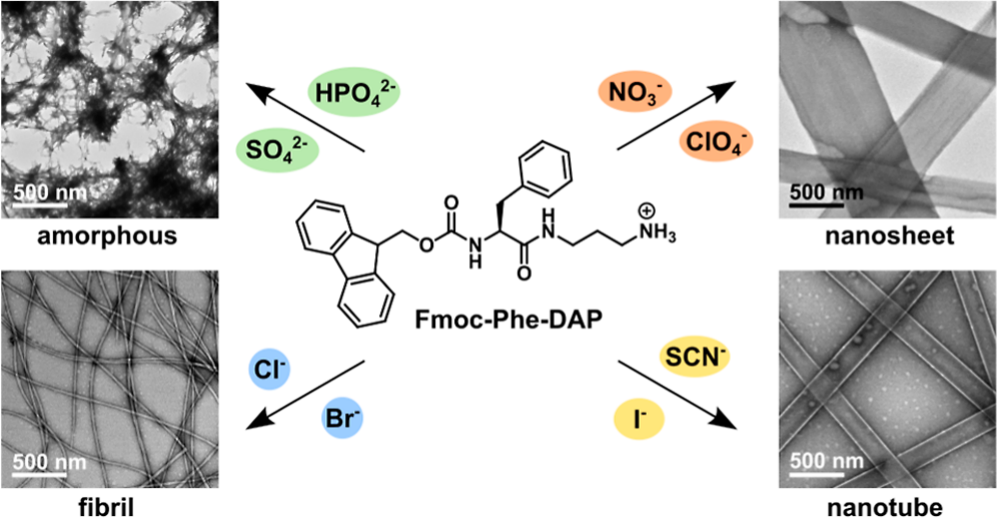

Supramolecular hydrogels have emerged as a class of promising biomaterials for applications such as drug delivery and tissue engineering. Self-assembling peptides have been well studied for such applications, but low molecular weight (LMW) amino acid-derived gelators have attracted interest as low-cost alternatives with similar emergent properties. Fluorenylmethyloxycarbonyl-phenylalanine (Fmoc-Phe) is one such privileged motif often chosen due to its inherent self-assembly potential. Previously, we developed cationic Fmoc-Phe-DAP gelators that assemble into hydrogel networks in aqueous NaCl solutions of sufficient ionic strength. The chloride anions in these solutions screen the cationic charge of the gelators to enable self-assembly to occur. Herein, we report the effects of varying the anions of sodium salts on the gelation potential, nanoscale morphology, and hydrogel viscoelastic properties of Fmoc-Phe-DAP and two of its fluorinated derivatives, Fmoc-3F-Phe-DAP and Fmoc-F_5_-Phe-DAP. It was observed that both the anion identity and gelator structure had a significant impact on the self-assembly and gelation properties of these derivatives. Changing the anion identity resulted in significant polymorphism of the nanoscale morphology of the assembled states that was dependent on the chemical structure of the gelator. The emergent viscoelastic character of the hydrogel networks was also found to be reliant on the anion identity and gelator structure. These results demonstrate the complex interplay between the gelator and environment that have a profound and often unpredictable impact on both self-assembly properties and emergent viscoelasticity in supramolecular hydrogels formed by LMW compounds. This work also illustrates the current lack of understanding that limits the rational design of potential biomaterials that will be in contact with complex biological fluids and provides motivation for additional research to correlate the chemical structure of LMW gelators with the structure and emergent properties of the resulting supramolecular assemblies as a function of environment.

## Introduction

Supramolecular hydrogels are dynamic materials that are useful for a variety of biomedical applications, including wound healing, antimicrobial therapy, drug delivery, and tissue engineering.^1−4^ While polymer hydrogels have often been utilized for such applications,^5−7^ self-assembling peptides can form hydrogels that are nearly ideal for these applications due to their inherent biocompatibility and bioactivity.^8−14^ The emergent properties of peptide-based supramolecular materials are also highly tunable through modification of the amino acid sequence,^15−17^ but a barrier to their widespread adoption is the high cost of production of synthetic peptides.^18−20^ As a result, low molecular weight (LMW) supramolecular gelators derived from amino acids have been investigated as cost-effective alternatives to peptide-derived gelators.^20−26^ A major challenge in the design of LMW supramolecular hydrogels is that subtle changes to the chemical structure of the gelator and to the environment often have profound and unforeseen effects on the emergent properties of the assembled materials.^26−29^

**Figure 1:**
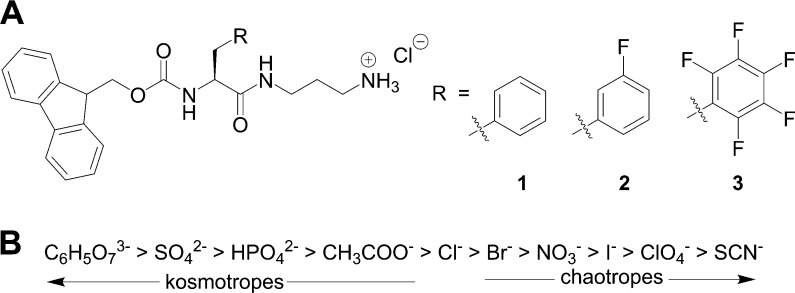
(A) Chemical structure of Fmoc-Phe-DAP derivatives: Fmoc-Phe-DAP (**1**), Fmoc-3F-Phe-DAP (**2**), and Fmoc-F_5_-Phe-DAP (**3**). (B) Hofmeister series of anions, listed in order from kosmotropic (left) to chaotropic (right).

Fluorenylmethyloxycarbonyl-phenylalanine (Fmoc-Phe) derivatives have been frequently leveraged as LMW gelators since they have proven to have a strong propensity to self-assemble into nanostructures that entangle to form hydrogel networks.^30−34^ While Fmoc-Phe has been shown to self-assemble into hydrogel networks, it has also been demonstrated that modification of the side-chain phenyl ring modulates gelation behavior and often results in improved emergent viscoelastic properties.^35−42^ For example, perfluorination of the phenyl ring (Fmoc-F_5_-Phe) and monofluorination at the meta position (Fmoc-3F-Phe) have been shown to produce hydrogel networks with superior viscoelastic rigidity and shear response properties compared to unmodified Fmoc-Phe.^41−45^ Modification of the C-terminal carboxyl group of the Fmoc-Phe scaffold has also been used to tune the gelation properties of Fmoc-Phe derivatives. Fmoc-Phe derivatives with unmodified C-termini are poorly soluble in water at neutral pH, necessitating solubilization of these derivatives using either an organic cosolvent or a high-pH aqueous solution.^33,45,46^ To overcome this limitation and create gelators more suitable for biological conditions, we have designed water-soluble Fmoc-Phe derivatives by conjugating diaminopropane to the C-terminus (**1**–**3**, Figure 1A).^47,48^ The terminal amine on these Fmoc-Phe-DAP derivatives is cationic at neutral pH, dramatically increasing the solubility of the gelators while also preventing immediate gelation due to charge repulsion of the appended cations. Rapid self-assembly and gelation of these derivatives is initiated by increasing the ionic strength of the solution by addition of saline, which contains chloride anions that screen the repulsive interactions of the cationic ammonium groups. As was found with Fmoc-Phe derivatives, fluorination or perfluorination of the Fmoc-Phe-DAP (**1**) phenyl ring [Fmoc-3F-Phe-DAP (**2**) and Fmoc-F_5_-Phe-DAP (**3**)] strongly impacts the emergent hydrogel properties, including viscoelastic storage and loss moduli (*G*′ and *G*″, respectively), as well as the nanoscale morphology of the assemblies formed by each gelator, with Fmoc-3F-Phe-DAP forming mixtures of nanofibrils and nanotubes and Fmoc-F_5_-Phe-DAP favoring nanofiber morphologies (Figure 1A).^47^

Varying the environmental conditions under which assembly occurs also affects the self-assembly and resultant emergent properties of LMW compounds.^45,46,49−51^ For example, assembling supramolecular gelators in the presence of different salts has been shown to impact a variety of material properties, including assembly morphology and viscoelastic *G*′/*G*″.^52−55^ The specific anions or cations of the salts used can sometimes produce effects that trend with their placement in the Hofmeister series.^56,57^ The Hofmeister series refers to the arrangement of anions or cations in order of their ability to decrease (kosmotropes) or increase (chaotropes) the solubility of proteins (Figure 1B) and was first discovered by Franz Hofmeister more than a century ago.^58,59^ Since then, the Hofmeister series has been found to affect numerous other phenomena, from enzyme activity^60,61^ to polymer hydrogel mechanics^62−65^ to supramolecular hydrogel viscoelasticity.^55−57^ Ulijn and co-workers showed that gelation of the dipeptide Fmoc-YL in the presence of different sodium salts led to hydrogels with variable viscoelasticity and morphology—kosmotropic anions produced stronger gels with elongated fibers and chaotropic anions produced weaker gels composed of spherical aggregates.^56^ More recently, Zhang et al. reported a similar trend in viscoelasticity when examining the gelation of a LMW d-gluconic acetal derivative in the presence of various anions.^57^ Since Fmoc-Phe-DAP derivatives form hydrogels by direct interaction of the cationic amine with the chloride anion of NaCl, we were curious how changing the anion would impact the morphology and viscoelasticity, and if any Hofmeister trends would be observed.

Consequently, we report herein the effect of 10 different sodium salts on the self-assembly and gelation, assembly morphology, and emergent viscoelastic moduli of Fmoc-Phe-DAP (**1**), Fmoc-3F-Phe-DAP (**2**), and Fmoc-F_5_-Phe-DAP (**3**). Surprisingly, we observed different effects of anion identity on the nanoscale and mesoscale properties of the assemblies for each of the three gelators. The unmodified Fmoc-Phe-DAP (**1**) gelator self-assembled into a variety of morphologies (fibrils, nanoribbons/nanotubes, and sheets) with a range of hydrogel properties as a function of added anion identity. Fmoc-3F-Phe-DAP (**2**) assembled into varied morphologies similar to Fmoc-Phe-DAP but exhibited viscoelastic moduli that were largely independent of the anion added. In contrast, Fmoc-F_5_-Phe-DAP (**3**) assembled into identical nanofibers regardless of the anion used, although the viscoelastic moduli were found to be dependent on anion identity. These findings illustrate the complex interplay between the gelator and the environment that have a strong and unpredictable impact on both self-assembly properties and emergent viscoelasticity in supramolecular hydrogels formed by LMW materials. This work also demonstrates the current lack of understanding that limits the rational design of potential biomaterials that will be in contact with complex biological fluids and provides motivation for additional research to correlate the chemical structure of LMW gelators with the structure and emergent properties of the resulting supramolecular assemblies as a function of environment.

## Experimental Section

### Materials

Reagents and organic solvents were purchased commercially and used without further purification. Compounds **1–3** were synthesized following previously reported protocols.^66^ Water used for gelation was purified using a nanopure filtration system (Barnstead NANOpure, 0.2 μm filter, 18.2 MΩ cm).

### Self-Assembly Conditions

All assemblies for the following experiments were prepared with a final gelator concentration of 10 mM, a final salt concentration of 100 mM, and a total volume of 2 mL except for critical gelation concentration experiments. Compounds **1–3** (0.02 mmol) were dissolved in 1.6 mL of water in a glass vial by heating in a 70 °C water bath for 1 min, followed by sonication for 30 s, and heating again for 30 s. Then, 0.4 mL of a 500 mM aqueous solution of a sodium salt was added to initiate self-assembly/gelation. Immediately after the addition of the salt solution, the vial was briefly agitated by a vortex mixer. Formation of hydrogels, viscous colloidal suspensions, or opaque precipitates was observed within seconds to minutes. To determine the critical gelation concentration of each gelator and salt combination, a stock solution corresponding to the highest gelator concentration (either 10 mM or 15 mM) was prepared. The stock gelator solution was then aliquoted and diluted with water to prepare 1.6 mL solutions for assembly such that after addition of 0.4 mL of a 500 mM sodium salt solution, assemblies with a total volume of 2 mL were formed that ranged from 1–10 mM or 10–15 mM in 1 mM increments. Digital images of all assemblies formed to determine the critical gelation concentration are provided in the Supporting Information (Figures S1–S6). Digital images of all 10 mM assemblies at 30 min, 24 h, and 1 week after salt addition are provided in the Supporting Information (Figures S7–S12).

### Transmission Electron Microscopy

Transmission electron microscopy (TEM) images were obtained using a Hitachi 7650 transmission electron microscope with an accelerating voltage of 80 kV. Aliquots of assembled materials (8 μL) were applied directly onto 200 mesh carbon-coated copper grids and allowed to stand for 1 min. Excess sample was carefully removed by capillary action using filter paper, and the grids were then stained with 2% (w/v) uranyl acetate (8 μL) for 2 min. Excess stain was again removed by capillary action, and the grids were allowed to air-dry for 10–15 min. Dimensions of the nanostructures were determined using ImageJ software and are reported either as the average of at least 100 independent measurements with error reported as the standard deviation about the mean value or as a range of observed values when nanostructures vary significantly in size (Tables S1–S3). TEM images of all assemblies at 30 min, 24 h, and 1 week after salt addition are provided in the Supporting Information (Figures S7–S12).

### Mass Spectrometry

Mass spectra were obtained on an Advion Interchim Scientific Expression compact mass spectrometer in a positive mode, coupled with an Agilent Life Sciences 1260 Infinity II VW Detector. Selected assemblies to be analyzed were prepared at 10 mM as described above, and 1 week after salt addition the samples were frozen and lyophilized. Following lyophilization, samples were resuspended in methanol to a final concentration of 1 mg/mL and filtered with a 0.2 μm nylon membrane filter before analysis. Unassembled gelators **1**–**3** were also dissolved in methanol at 1 mg/mL for analysis and comparison. Mass spectra of the unassembled gelators and chosen assemblies are provided in the Supporting Information (Figures S13–S24).

### NMR Spectroscopy

NMR spectra were obtained using a Brüker Avance 500 MHz spectrometer. ^1^H chemical shifts on reported spectra are with reference to TMS at 0 ppm. NMR spectra of compounds **1–3** and selected assemblies with nitrate and perchlorate which were frozen, lyophilized, and resuspended in DMSO-*d*_6_ are provided in the Supporting Information (Figures S25–S27). ^1^H NMR was also used to determine monomer concentration in selected assemblies. Compounds **1–3** (0.02 mmol) were dissolved in 0.8 mL of D_2_O in a glass vial by heating and sonication as described above. Then, this solution was placed into an NMR tube and 0.2 mL of a 500 mM aqueous solution of sodium salt prepared in D_2_O was added, and the tube was agitated by a vortex mixer. Reference solutions of compounds **1–3** at 10 mM in DMSO-*d*_6_ and D_2_O without added salt were prepared as controls for unassembled and partially assembled states. NMR tubes were fitted with an internal capillary containing 24 mM DMF in DMSO-*d*_6_ as an external standard. The percent of unassembled monomer was measured by comparative integration of signal peaks in the aromatic region of the DMSO-*d*_6_ sample for each compound. Each signal was integrated relative to the external DMF standard. NMR spectra of compounds **1–3** and selected assemblies used for monomer experiments are provided in the Supporting Information (Figures S37–S39).

### Oscillatory Rheology

Rheological measurements were obtained using a TA Instruments Discovery HR-2 rheometer. A 20 mM parallel plate geometry was used for the experiments. Hydrogels of 1 mL volume were formed in 1.5 mL plastic microcentrifuge tubes. Immediately before rheological characterization, the plastic tube containing the hydrogel was cut at the 0.5 mL line using a razor blade and the cylindrical hydrogel was placed directly onto the Peltier plate for characterization. Experiments were performed using an average gap size of 1.2 mm operating in an oscillatory mode. Strain sweep experiments were performed from a 0.01–100% strain at a constant frequency of 6.283 rad s^–1^ to determine the linear viscoelastic region for each hydrogel. All strain sweep data can be found in the Supporting Information (Figures S30–S32). Frequency sweeps were performed from 0.1–100 rad s^–1^ at a constant strain of 0.2%, which falls within the linear viscoelastic region for all hydrogels examined. Values at the upper end of the frequency sweep were cut off when the raw phase angle increased above 175° as recommended for the TA DHR series of rheometers, since values beyond this point are dominated by the instrument inertial torque instead of the sample torque.^67^ Reported values for storage and loss moduli (*G*′ and *G*″, respectively) are the average of at least three distinct measurements on separate hydrogels with the error reported as the standard deviation about the mean (Table S5). All frequency sweeps can be found in the Supporting Information (Figures S33–S35).

## Results and Discussion

### Self-Assembly of Fmoc-Phe-DAP Derivatives Using a Series of Sodium Salts with a Range of Anions

We have previously characterized the self-assembly of Fmoc-Phe-DAP (**1**) and the fluorinated derivatives Fmoc-3F-Phe-DAP (**2**) and Fmoc-F_5_-Phe-DAP (**3**) using NaCl as an initiation agent.^47,48^ These Fmoc-Phe-DAP derivatives are soluble in water due to the cationic terminal amine but will not form a hydrogel until NaCl is added to the solution to screen the charges and allow the molecules to pack together into a supramolecular network. The hydrogels of **1–3** self-assembled in the presence of NaCl exhibit varying turbidity, viscoelasticity, and substructure morphology. Others have reported that emergent viscoelastic storage and loss moduli of other supramolecular hydrogels formed in the presence of varying salts follow a decreasing trend along the Hofmeister series, where assemblies with the most kosmotropic anions have the highest moduli and assemblies with the most chaotropic anions have the lowest moduli.^56,57^ Based on the observation that chloride anions in NaCl play a central role in initiating self-assembly and gelation of Fmoc-Phe-DAP compounds, we hypothesized that self-assembly and gelation of these derivatives would also be sensitive to the anion identity of sodium salts. The work reported herein interrogates this hypothesis by examining the effects of anion identity on the emergent self-assembly and hydrogel viscoelastic character of the corresponding assemblies.

**Figure 2:**
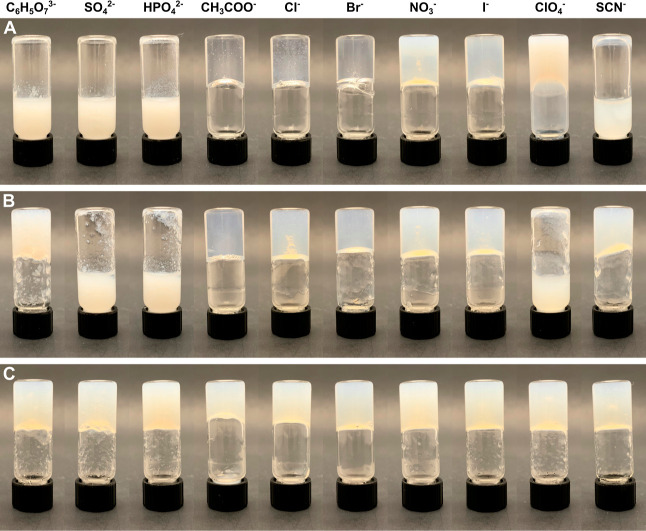
Digital images of assemblies of (A) Fmoc-Phe-DAP (**1**), (B) Fmoc-3F-Phe-DAP (**2**), and (**C**) Fmoc-F_5_-Phe-DAP (**3**) taken 30 min after addition of the indicated sodium salts to solutions of each gelator. Each anion used is listed at the top of the corresponding columns.

Accordingly, we assessed self-assembly of compounds **1–3** with the sodium salt of each anion shown in Figure 1B. Each derivative was dissolved by heating and sonication of the solution; then, an aqueous solution of sodium salt was added to the vial to trigger rapid self-assembly. All assemblies had a constant final gelator concentration of 10 mM and final salt concentration of 100 mM to enable direct comparison between all samples (Figure 2). It should be noted that compounds **1–3** were synthesized and obtained as the chloride salt, but any impact by this counterion in the assemblies should be negligible since the salt solutions used to initiate assembly have a 10-fold excess of added anion. Digital images of Fmoc-Phe-DAP (**1**) assemblies taken 30 min after salt addition provide clear evidence that the anion identity can affect both the gelation ability of **1** and the turbidity of the resulting assemblies (Figure 2A). These samples can be grouped into three categories based on visual inspection. The kosmotropic anions (citrate, sulfate, and hydrogen phosphate) caused **1** to form a white precipitate immediately upon mixing. Addition of chloride and the two “borderline” anions, acetate and bromide, to solutions of **1** triggered the formation of transparent hydrogels. Lastly, mixing **1** with the four chaotropic anions (nitrate, iodide, perchlorate, and thiocyanate) produced turbid or opaque assemblies. Hydrogels were formed using all chaotropic salts except thiocyanate, which instead resulted in a turbid colloidal suspension.

Since compound **1** displayed a wide range of assembly behavior at a constant gelator concentration of 10 mM when assembled using different sodium salts, we determined the critical gelation concentration (CGC) of **1** with each sodium salt (Table 1 and Figures S1 and S2). Samples that formed self-supporting hydrogels at 10 mM were assembled with each anion between 1 and 10 mM gelator concentration to determine the CGC, and samples that did not form hydrogels at 10 mM were assembled between 10 and 15 mM (Figures S1 and S2). The CGC of **1** when assembled with the different sodium salts was found to be variable, depending on the anion, similar to the variable gelation behaviors observed at constant gelator concentration. No CGC was found for **1** when assembled with the kosmotropic anions (citrate, sulfate, and hydrogel phosphate), since a white precipitate was formed at all gelator concentrations tested (Table 1 and Figure S1A–C). The lowest CGC of 3–4 mM was observed for **1** when assembled with acetate or chloride, which are two anions from the borderline group, though a higher CGC of 9–10 mM was found for assembly with bromide (Table 1 and Figure S2A–C). Assembly of **1** with the four chaotropic anions (nitrate, iodide, perchlorate, and thiocyanate) resulted in relatively higher CGC values, ranging from 7–8 mM for the nitrate sample to 14–15 mM for the thiocyanate sample (Table 1 and Figures S1D and S2D–F).

**Table 1:** Critical Gelation Concentration for Each Gelator **1–3** in the Presence of Different Sodium Salts^a^

	Critical Gelation Concentration (mM)		
Salt	Fmoc-Phe-DAP (1)	Fmoc-3F-Phe-DAP (2)	Fmoc-F_5_-Phe-DAP (3)
Na_3_C_6_H_5_O_7_	–	4 to 5	5 to 6
Na_2_SO_4_	–	11 to 12	2 to 3
Na_2_HPO_4_	–	11 to 12	1 to 2
NaOAc	3 to 4	1 to 2	1 to 2
NaCl	3 to 4	3 to 4	5 to 6
NaBr	9 to 10	2 to 3	4 to 5
NaNO_3_	7 to 8	4 to 5	2 to 3
NaI	9 to 10	4 to 5	1 to 2
NaClO_4_	9 to 10	10 to 11	2 to 3
NaSCN	14 to 15	4 to 5	4 to 5

a Values are reported as a range between the highest concentration that does not form a hydrogel stable to vial inversion and the lowest concentration that forms a partial or full hydrogel stable to vial inversion. Where values are not reported, samples did not gel under any condition tested up to the maximum 15 mM gelator concentration.

Interestingly, assemblies of the fluorinated gelators **2** and **3** at 10 mM did not follow the same pattern as assemblies of **1** based on visual inspection alone (Figure 2). Addition of citrate to a solution of **2** supported the formation of an opaque hydrogel, while the other kosmotropic ions, sulfate and hydrogen phosphate, caused precipitation similar to what was observed with these salts for compound **1** (Figure 2B). Hydrogels formed by **2** in the presence of the borderline group of anions (acetate, chloride, and bromide) were more turbid than those formed by **1** under these conditions. For the chaotropic anions, the observed gelation pattern for **2** was swapped in the presence of perchlorate and thiocyanate when compared to **1**, but turbid hydrogels were still formed with nitrate and iodide. Lower CGC values were generally found for assemblies of **2** with each anion when compared to **1**, apart from samples assembled with perchlorate (Table 1). Assembly of **2** with the kosmotropes produced variable CGC values, with citrate providing a lower CGC of 4–5 mM, while sulfate and hydrogen phosphate only supported gelation above 11 mM of gelator **2** (Table 1 and Figures S4A and S3A,B). CGC values for assemblies of **2** with the borderline anion group of acetate, chloride, and bromide were lower and less variable, ranging from 1–4 mM (Table 1 and Figure S4B–D). Lastly, high variability in CGC similar to that observed for kosmotropic anions was found when **2** was assembled with chaotropic anions, with a lower CGC of 4–5 mM determined for nitrate, iodide, and thiocyanate but a higher CGC of 10–11 mM observed for perchlorate (Table 1 and Figures S3C and S4E–G). In contrast to the assemblies of compounds **1** and **2**, the perfluorinated gelator **3** formed visually similar opaque hydrogels at 10 mM upon mixing with all ten sodium salts (Figure 2C). Some variability was observed in the CGC of **3** with each anion, but all values were found to be within 1–6 mM and did not appear to follow any trend based on the Hofmeister series order (Table 1 and Figures S5 and S6). It is evident that fluorination at the phenyl side chain of Fmoc-Phe-DAP gelators affects their supramolecular architecture enough that the same anions can produce different gel states and CGC values for different derivatives. To interrogate these differences further, nanoscale morphologies of these assemblies were investigated.

### Morphology of Fmoc-Phe-DAP Derivative Assemblies

Negative-stain TEM was used to characterize the morphology of the self-assembled structures within each sample. Each sample was imaged at 30 min, 24 h, and 1 week after addition of the salt, since this class of gelators is known to undergo a hierarchical assembly process over time depending on the conditions.^47,48,66^ Select images of samples at the 30 min time point are shown herein to illustrate the effects of anion identity on assembly, with any differences observed after longer time periods discussed in the text. The entire collection of images for all samples and time points can be found in the Supporting Information (Figures S7–S12).

**Figure 3:**
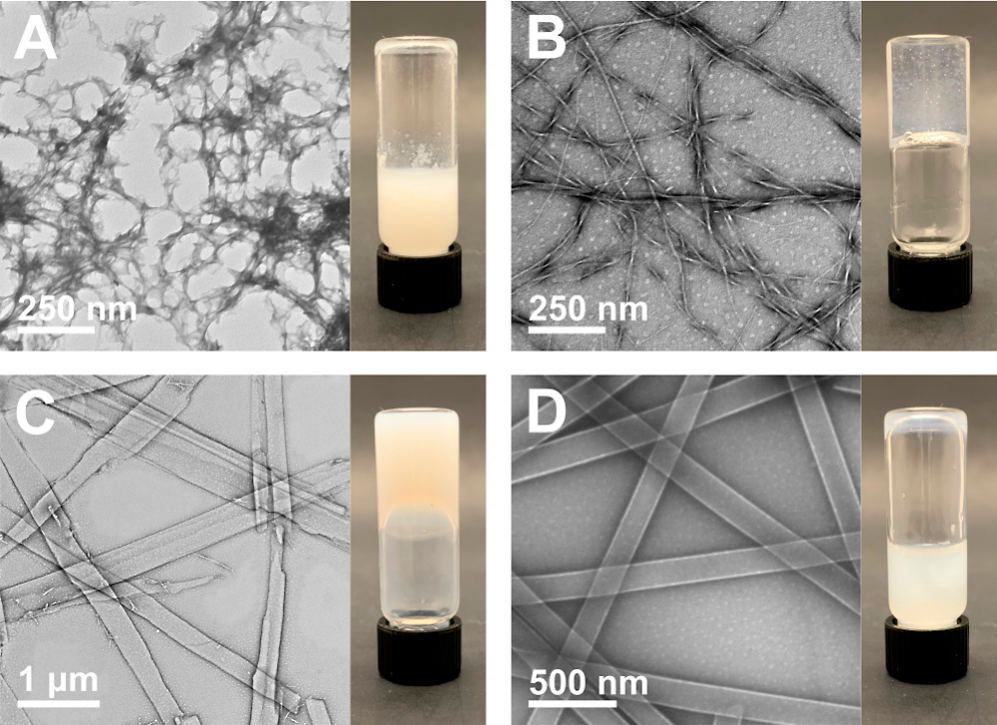
TEM and digital images of Fmoc-Phe-DAP (**1**) assemblies 30 min after addition of sodium salts of (A) citrate, (B) chloride, (C) perchlorate, and (D) thiocyanate.

TEM analysis of Fmoc-Phe-DAP (**1**) assemblies revealed a diverse set of morphologies induced by the different sodium salts (Figures 3 and S7,S8). Four different classes of assembly were observed that can be linked to the three anion groups discussed in the previous section: kosmotropes, borderline ions, and chaotropes. A split in behavior was observed in samples containing chaotropic anions, so these ions were split into two groups, resulting in four total groups. The precipitate formed by the kosmotropic anion group is an amorphous aggregate, as seen in the citrate sample (Figures 3A and S7A) and the sulfate and hydrogen phosphate samples (Figure S7D,G). These samples remain as amorphous-appearing aggregates after 1 week (citrate: Figure S7B,C, sulfate: Figure S7E,F, hydrogen phosphate: Figure S7H,I). To determine if this behavior was a result of the higher ionic strength of these solutions due to the polyvalent anions, assemblies of **1** were also prepared with a final concentration of 16.7 mM sodium citrate, 33.3 mM sodium sulfate, and 33.3 mM sodium hydrogen phosphate, and identical precipitation was observed.

Samples assembled using the three borderline anions, acetate, chloride, and bromide, exhibit hierarchical assembly over time, as we have previously observed for hydrogels of **1** formed with chloride anions (Figures S7J–O, and S8A–C).^47,48,66^ Generally speaking, these samples are composed primarily of fibrils with a width of 5–8 nm after initial assembly (Figure 3B, Table S1, and Figures S7J,M and S8A), followed by the appearance of twisted nanoribbons after a period of hours that range in width from 6 to 450 nm (Table S1 and Figures S7K,N and S8B), which finally mature into nanotubes after a day or more that are 187–600 nm wide (Table S1 and Figures S7L,O and C). This evolution from twisted nanoribbons to nanotubes over time is associated with an observed increase in the turbidity of the hydrogels at the macroscale presumably due to increased light scattering by the larger nanotube structures. Only slight differences were observed in the apparent distribution of fibrils, nanoribbons, and nanotubes over time between the acetate, chloride, and bromide samples (Table S1 and Figures S7J–O and S8A–C).

Lastly, the chaotropic anion group showed two distinct dominant morphologies based on anion identity (Figure S8D–O). The dominant morphology of assemblies formed in the presence of the nitrate and perchlorate oxyanions were irregular, pseudo-crystalline nanosheets with widths of 38–487 nm and 88–2223 nm, respectively (Figure 3C and Figures S8D–F and J–L); fibrils 10.7 ± 1.2 nm wide were also observed in the nitrate sample (Table S1). The initial self-supporting perchlorate hydrogel transformed over time into a non-self-supporting colloidal suspension (Figure S8J–L); so, a high proportion of these large, irregular nanosheets will likely destabilize the hydrogel network. The other chaotropes, iodide and thiocyanate, induced immediate formation of nanotubes that were 210 ± 23 nm and 165 ± 20 nm wide, respectively (Figure 3D, Table S1, and Figures S8G–I and M–O). We hypothesize that the difference in morphologies in the chaotropic anion group stems from how the shape of the ions influences interaction with the Fmoc-Phe-DAP derivatives. For example, the oxyanions may support multiple distinct contact points with the cationic Fmoc-Phe-DAP assemblies, facilitating formation of the more extensive two-dimensional nanosheet structures.

**Figure 4:**
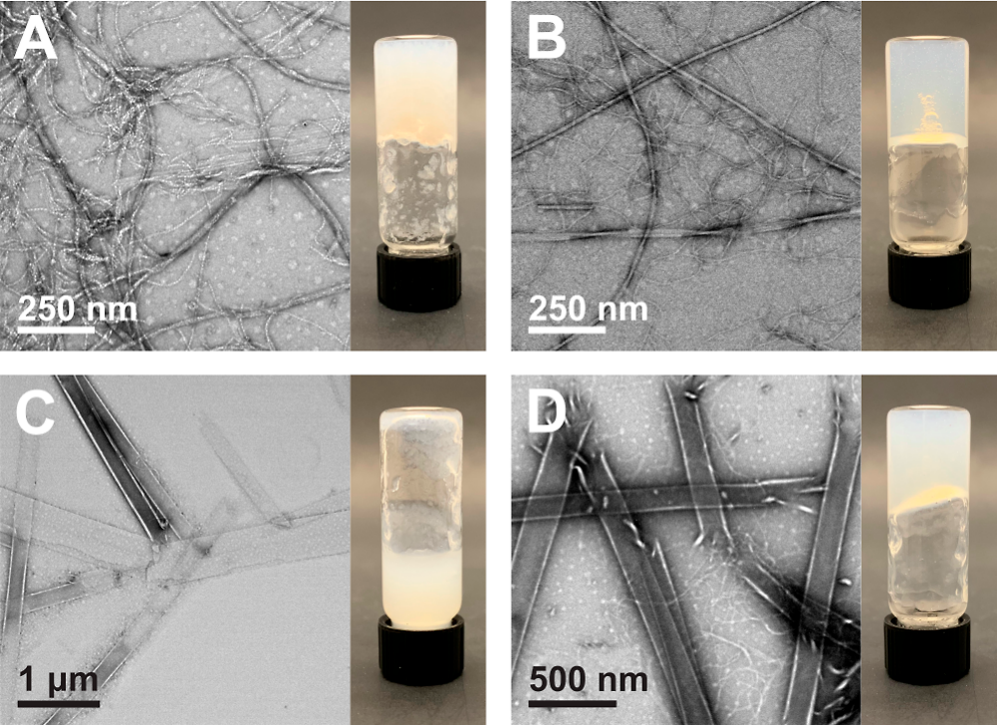
TEM and digital images of Fmoc-3F-Phe-DAP (**2**) assemblies 30 min after the addition of sodium salts of (A) citrate, (B) chloride, (C) perchlorate, and (D) thiocyanate.

The monofluorinated Fmoc-3F-Phe-DAP (**2**) derivative exhibited different morphological self-assembly patterns in the presence of the various anions than were observed with **1**. In contrast to the amorphous aggregates observed with **1**, an assortment of fibrils 14.5 ± 3.5 nm wide were observed in the citrate hydrogel of **2**, which fused into larger, twisted fibers with a width of 27.0 ± 5.7 nm after a week (Figure 4A, Table S2, and Figure S9A–C). Nanofibrils that were 9.4 ± 1.3 nm wide were observed in the sulfate sample initially (Table S2 and Figure S9D), which also fused into larger twisted fibers 27.6 ± 5.1 nm wide after 24 h (Table S2 and Figure S9E–F), while the hydrogen phosphate sample remained as an amorphous aggregate after 1 week (Figure S9H–I); neither anion supported the formation of a self-supporting hydrogel network (Figure S9D–I). Assemblies of **2** with lower anion concentrations to match the ionic strength of the samples with monovalent salts were again prepared as described for samples of **1**, and again no morphological differences were observed.

Some deviation in the borderline anion group is also observed when comparing the hierarchical assembly of gelators **1** and **2**. The fluorinated gelator **2** forms fibrils with widths of 6.1 ± 0.7 nm or 5.5 ± 0.9 nm initially when assembled using chloride or bromide, respectively (Figure 4B, Table S2, and Figures S9M,N and S10A,B), but nanoribbons 76–129 nm wide and a few nanotubes 57–109 nm wide are only observed 1 week after assembly (Table S2 and Figures S9O and S10C). In contrast, the acetate hydrogel displayed a mix of fibrils with a width of 11.1 ± 1.6 nm and nanotubes with a width of 198.7 ± 24.6 nm initially, and these morphologies were consistently observed after 24 h and 1 week (Table S2 and Figure S9J,L). These findings may indicate that the different ion shape of the acetate anion compared to the spherical chloride and bromide ions could be directly impacting the morphological outcome of assembly in the case of **2**, since nanotubes were formed much more rapidly in the acetate assemblies.

Incubation of **2** with the chaotropic oxyanions, nitrate and perchlorate, resulted in similar morphological outcomes as observed for assemblies of **1**. Irregular nanosheet structures with widths of 112–1018 nm and 45–1039 nm were observed for assemblies with nitrate and perchlorate, respectively, with nitrate assemblies also containing fibrils with a width of 6.3 ± 0.9 nm (Figure 4C, Table S2, and Figures S10D–F and J,L). Interestingly, nanotubes 202.7 ± 15.0 nm wide were also observed in the non-gel perchlorate sample at the initial 30 min time point, but the irregular nanosheets became the dominant morphology after 1 week (Figure 4C, Table S2, and Figure S10J,L). For the remaining chaotropes, iodide and thiocyanate, primarily nanotubes that were 194.1 ± 17.9 nm and 189.8 ± 26.7 nm wide, respectively, were observed (Figure 4D, Table S2, and Figures S10G,I and M–O). Fibrils with a width of 17.0 ± 4.2 nm were also present in the iodide sample initially, but nanotubes were the dominant morphology for both samples after 1 week (Table S2, and Figures S10G–I and M–O).

When comparing the morphological outcomes of self-assembly of **1** and **2**, it is apparent that varying the anion produced similar, but not identical, effects. The same four groupings of self-assembly behavior were observed, but subtle morphological or gelation state differences existed in each group. For example, the kosmotrope citrate supported the formation of a hydrogel from solutions of **2** but not solutions of **1** (Figures 3A and 4A). The borderline anions exhibited split behavior, since acetate accelerated the formation of nanotubes of **2** compared to samples of **1** (Figures S7J,L and S9J,L), but a slower transition to nanotubes was observed in chloride and bromide samples of **2** compared to samples of **1** (Figures S7M–O, S8A–C, S9M–O and S10A–C). Additionally, the nanotubes formed by **2** when incubated with the borderline anions were in the much smaller range of 100–200 nm compared to nanotubes formed by samples of **1**, which varied from less than 200 nm to about 600 nm wide (Tables S1 and S2). Lastly, the chaotropic anions induced the formation of multiple morphologies for each anion at initial time points in samples of **2**, whereas samples of **1** often contained one dominant morphology of either nanotubes or nanosheets for each anion (Figures S8D–O and S10D–O). A complex interplay between the effects of chemical structure differences and the effects of environmental differences of the various anions influences the self-assembly of these gelators, which can be difficult to unravel from each other. Generally, it appears that the addition of fluorine in the side chain of **2**, which results in slightly increased hydrophobicity and altered phenyl ring electronics compared to **1**, causes a slower hierarchical assembly process in most cases. This may indicate that the effects of gelator chemical structure in the case of **2** are starting to override the environmental influences of the anions when compared to the unmodified chemical structure of gelator **1**.

**Figure 5:**
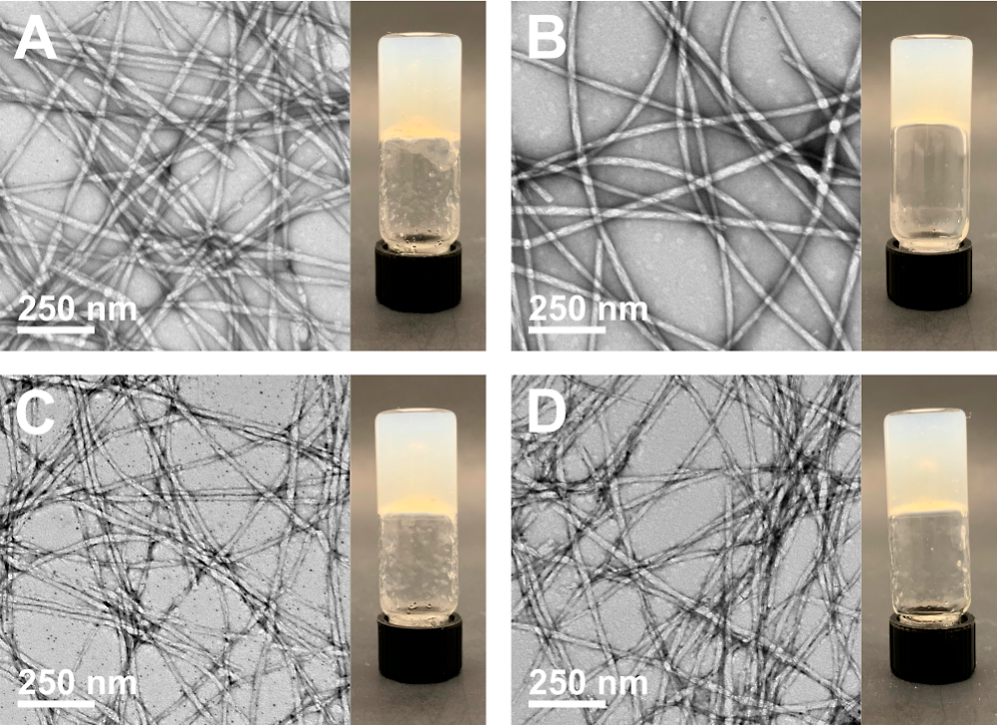
TEM and digital images of Fmoc-F_5_-Phe-DAP (**3**) assemblies 30 min after addition of sodium salts of (A) citrate, (B) chloride, (C) perchlorate, and (D) thiocyanate.

Interestingly, TEM analysis of all hydrogels of Fmoc-F_5_-Phe-DAP (**3**) indicated that this molecule assembled into identical nanofibers regardless of anion identity (Figure 5 and Figures S11 and S12). This outcome agrees with the observation that the anion identity had little influence on the formation of self-supporting hydrogels (Figure 2C). In all cases, TEM analysis indicated that the hydrogels were composed only of nanofibers with widths ranging from 20–29 nm (Table S3). Additionally, the nanofiber morphology in hydrogels of **3** remained unchanged over 1 week of observation, unlike the assemblies of **1** and **2**, which were found to evolve over time in many cases. The self-assembly properties of Fmoc-F_5_-Phe-DAP (**3**) demonstrate the complex interplay between the inherent properties of the gelator structure and the effect of the environment in influencing self-assembly pathways and the emergent properties of the assembly. The perfluorinated phenyl side chain of **3** dramatically changes both the electronics and the hydrophobicity of this derivative. These alterations in the properties of Fmoc-F_5_-Phe-DAP (**3**) result in a molecule in which the side-chain interactions dominate environmental effects in the self-assembly process.

Most of the morphologies formed by the various combinations of gelator and anions are consistent with the nanofibrils, nanoribbons, and nanotubes previously reported to form when assembling **1–3** by addition of chloride.^47^ However, the irregular nanosheets formed by **1** and **2** in the presence of the oxyanion chaotropes, nitrate and perchlorate, had not been previously observed in assemblies of Fmoc-Phe-DAP derivatives. Since the conjugate acids of these two anions (nitric acid and perchloric acid) are strong oxidizers, we further investigated the assemblies of **1–3** with nitrate and perchlorate to determine if oxidation of the gelators could explain the difference in morphology. All assemblies prepared with gelators **1–3** were examined after 1 week using mass spectrometry, ^1^H NMR, and reverse-phase HPLC (Figures S13–S30). No significant evidence of higher molecular mass oxidized products was observed using mass spectrometry when compared to samples of **1–3** alone or assembled with chloride as a control (Figures S13–S24), ^1^H NMR spectra of assembled nitrate and perchlorate samples were consistent with spectra of chloride samples and unassembled controls (Figures S25–27), and no shift in retention time of the gelator peak was observed by HPLC across assembled or unassembled samples for each gelator (Figures S28–S30). Therefore, oxidation of the gelators when assembled with nitrate and perchlorate was ruled out as an explanation for the irregular nanosheet morphologies formed by these samples.

### Viscoelastic Properties of Fmoc-Phe-DAP Derivative Hydrogels

**Figure 6:**
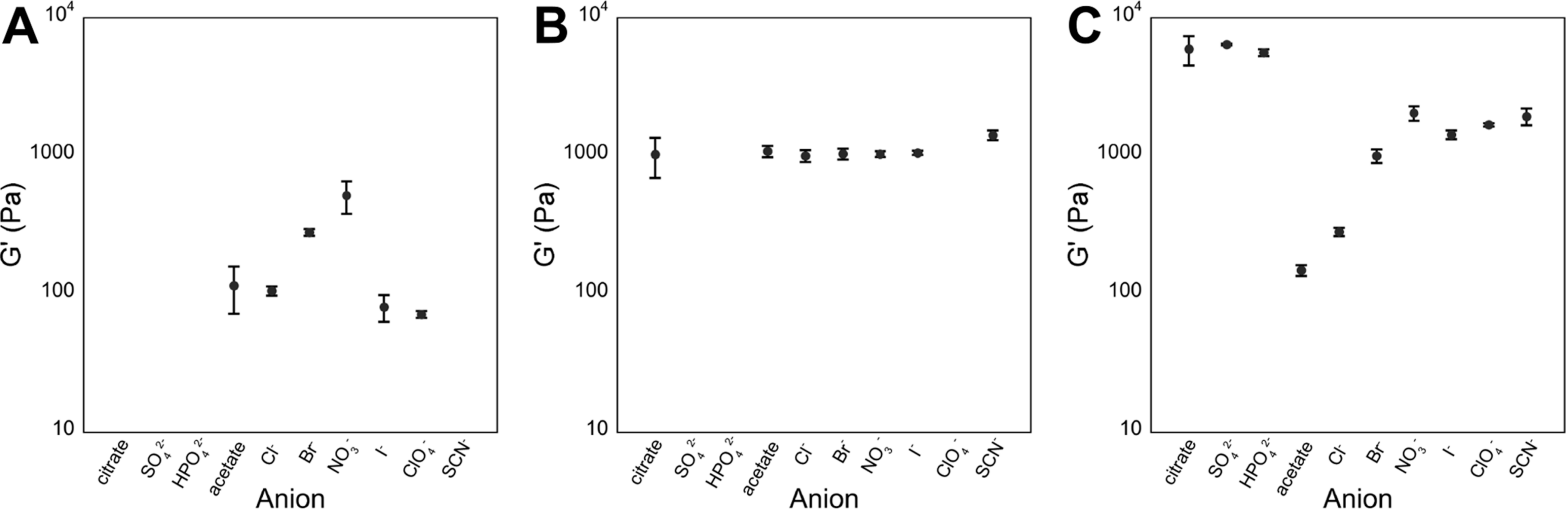
Average *G*′ values (Pa) taken from triplicate frequency sweep analysis of all samples that were hydrogels after 24 h plotted against the salt used to trigger assembly. Error reported is the standard deviation of the mean value. (A) Fmoc-Phe-DAP (**1**) hydrogels. (B) Fmoc-3F-Phe-DAP (**2**) hydrogels. (C) Fmoc-F_5_-Phe-DAP (**3**) hydrogels.

To investigate the environmental impact of anion identity on the emergent viscoelastic properties of resulting hydrogels, all samples that formed stable hydrogels after 24 h were examined using oscillatory rheology. Strain sweep measurements were performed on all hydrogel samples to ascertain the linear viscoelastic range for each material (Figures S31–S33). Then, frequency sweep experiments were performed on each sample under constant strain to determine the storage modulus (*G*′) and loss modulus (*G*″) as a function of angular frequency (Figures S34–S36). Generally, *G*′ and *G*″ were found to be parallel and separated by roughly an order of magnitude, indicating the presence of a structurally robust hydrogel state.^68^ The average storage and loss moduli for each sample were determined from triplicate frequency sweeps (Table S5). To convey the general trends observed, the average *G*′ value for each sample was plotted for each gelator as a function of the range of salts used to trigger assembly (Figure 6). Note that samples that failed to form self-supporting hydrogels lack data points in these representations.

It is abundantly clear upon examining the rheological data that the observed moduli trends cannot be rationalized entirely by the anion identity or assembly morphology alone. Markedly different behavior is observed for the three different gelators. No clear trend in *G*′ emerged for the six hydrogels of **1**, which ranged from a low value of 71 ± 4 Pa when gelled with perchlorate to a high value of 515 ± 136 Pa when gelled with nitrate (Figure 6A and Table S5). Notably, these two samples shared a similar sheet-like morphology, though the additional presence of fibrils in the nitrate assembly could explain the increased hydrogel strength. Two of the borderline anions, acetate and chloride, produced weak hydrogels with a *G*′ value comparable to the iodide hydrogel, which was made of predominantly nanotubes. However, when **1** was gelled using bromide the *G*′ values were over double that of the other borderline anions, despite sharing a similar polymorphic makeup of fibrils, nanoribbons, and nanotubes. Thus, while we can generalize about the stabilizing or destabilizing effect that certain morphologies have on viscoelastic properties of the resultant hydrogel network to an extent, specific interactions stemming from the anion identity likely also play a role in the overall behavior.

In the case of fluorinated gelators **2** and **3**, the trends that emerged were counterintuitive to what one might expect based on the observed morphologies. The assemblies of Fmoc-F_5_-Phe-DAP (**3**) were uniformly made up of nanofibers of similar dimensions regardless of the anion identity. Thus, it would be reasonable to predict that the hydrogels of **3** should have similar storage and loss moduli for each sample. In contrast, the morphology variability observed for Fmoc-3F-Phe-DAP (**2**) gels would be expected to give rise to a high degree of variability in the emergent hydrogel viscoelasticity as the network should be sensitive to the morphology of structures that comprise the network. Instead, it was observed that hydrogels of **2** had *G*′ values clustered closely around 1000 Pa except for the thiocyanate sample, which had a slightly higher modulus of 1421 ± 114 Pa (Figure 6B and Table S5). This was especially surprising considering the high degree of polymorphism seen with different sodium salts. The degree of polymorphism of assemblies of **1** and **2** was similar; hydrogels of **1** displayed highly variant viscoelasticity, whereas hydrogels of **2** were highly similar in viscoelasticity. Conversely, variable viscoelasticity was observed for hydrogels of **3** despite all ten samples displaying nearly identical assembly morphology (Figure 6C and Table S5). When the kosmotropic salts were used to gel **3**, a higher *G*′ value of approximately 6000 Pa was observed for all three samples. This may be a result of these three anions being polyvalent, thus allowing more potential bridging points between fibers to strengthen the network. For the rest of the monovalent salts in the borderline and chaotropic groups, the *G*′ values generally increased from left to right across the Hofmeister series (Figure 1B) except for nitrate, which had the highest storage modulus of this group (2054 ± 249 Pa). Again, this investigation into the viscoelastic properties of these hydrogels underscores that the gelator structure and the environment (anion identity) both have an impact on the emergent viscoelasticity. The correlation between these effects appears to be idiosyncratic in these studies, illustrating our lack of understanding regarding how each of these components collectively influence the emergent properties of these materials.

To probe for additional environmental effects on self-assembly stemming from possible buffering effects of the various anions, the pH of each assembly was measured at 30 min, 24 h, and 1 week after addition of salt (Table S6). For each sample, the variation in pH measured at different time points was small, less than 0.5 pH units for all except the assembly of **1** with perchlorate, which still varied by less than 1 pH unit. The most notable difference in the pH values of the samples was between the kosmotropic anions, which are all polyvalent, and the rest of the monovalent anions. The pH ranges for assemblies with the kosmotropes were 8.0–9.2 for samples of **1**, 7.3–9.0 for samples of **2**, and 6.5–8.8 for samples of **3**. At the higher end of these pH ranges, namely the hydrogen phosphate samples where the pH value approaches 9, it is possible that the deprotonation of some of the cationic ammonium groups in the gelators could be affecting the electrostatically modulated self-assembly process. However, we previously reported that compounds **1** and **2** form fibrils even at an alkaline pH value of 10.5 when assembled using NaCl but do not proceed through the hierarchical assembly process to form nanoribbons or nanotubes.^47^ Thus, we concluded that specific anion identity effects are outweighing any effects that may arise from a higher pH in the hydrogen phosphate assemblies of **1** and **2**, since the morphology observed was primarily amorphous aggregate and not distinct fibrils. Assemblies with monovalent anions had pH ranges of 6.1–7.0 for samples of **1**, 7.0–7.3 for samples of **2**, and 5.5–7.1 for samples of **3**. These near-neutral pH values are not expected to exert any significant effect on the self-assembly of compounds **1–3**, since the gelators should be essentially fully protonated at these pH values.

Lastly, the amount of monomer present in assemblies of different representative anions was determined to investigate if the anions impacted self-assembly propensity and if the observed effects on self-assembly and emergent assembly properties could be correlated with monomer concentration. ^1^H NMR spectra of each gelator **1–3** were collected in DMSO-*d*_6_ as unassembled monomeric controls, in D_2_O as the gelator solution prior to salt addition, and in D_2_O with representative anions as assembled samples (kosmotrope = citrate, borderline = chloride, oxyanion chaotrope = perchlorate, and chaotrope = thiocyanate) (Figures S37–S39). All of the gelators show partial self-assembly in aqueous solution without added salt, as the peaks in the aromatic region corresponding to the Fmoc and phenyl ring hydrogens displayed significant line broadening along with a reduction in the relative integration compared to an external standard, that is, features that are characteristic of self-assembled materials in solution-state NMR. Compound **1** contained the highest amount of monomer in the pre-salt solution at 63%, while the gelator **2** solution contained 48% monomer, and the gelator **3** solution contained the lowest amount of monomer at 14% (Figures S37B, S38B, and S39B). These results trend with the relative hydrophobicity of the three gelators, since gelator **1** is the least hydrophobic compound and has the most freely soluble monomer, while gelator **3** is the most hydrophobic derivative and has the least freely soluble monomer in water alone. Upon addition of the representative salts, very little monomer remained, regardless of gelator or anion identity with the exception of gelator **2** and chloride, where 12% monomer remained by relative integration of a single broad signal (Figure S38C). In all other cases, the remaining monomer concentration was ≤4% (Figures S37C–F, S38D–F, and S39C–F). Therefore, it is not likely that these small differences in monomer concentration after assembly with various anions are responsible for the observed effects on gelation potential, nanoscale morphology, or viscoelastic moduli.

### Discussion

Collectively, the results gathered here underscore the intricacies of supramolecular hydrogel systems and how subtle modifications to the system can drastically impact both self-assembly and emergent viscoelasticity in an unpredictable fashion. Gelators **1**–**3** displayed different patterns of behavior for hydrogel formation, nanoscale morphology, and viscoelastic moduli in each case; so, it is clear that the gelator structure greatly influences supramolecular organization as well as specific interactions with each anion. However, it is unclear if these behaviors arise from the electronic or steric differences in the phenyl rings due to the presence or absence of fluorines, the difference in overall hydrophobicity of the compounds, or a combination of all factors. The electronics of the ring are likely to have a strong impact on the supramolecular packing of the gelators and subsequent emergent material properties, since a driving force for assembly of Fmoc-Phe-derived gelators is the intermolecular interaction between neighboring aromatic fluorenyl ring systems and/or side-chain phenyl rings. For example, both gelator **1** and **3** have symmetric ring electronics, while gelator **2** has a distinct dipole resulting from the single fluorine. Since both gelators **1** and **3** showed variation in viscoelastic moduli, it is possible that this ring configuration affects the supramolecular organization and interactions in a way that allows the environmental effects of the specific anions to dominate and influence the overall network strength more directly. In contrast, the asymmetric electronics of gelator **2** may not allow such anion environmental effects to dominate the viscoelastic properties of the system. Another case to consider is the morphological homogeneity of all hydrogels of **3** when compared to gelators **1** and **2**. The perfluorinated ring of gelator **3** significantly alters the phenyl ring electronics by inverting the quadrupole moment of the aromatic ring, which may impact the molecular packing between gelator molecules.^69^ Additionally, anion−π interactions are well known to occur in highly fluorinated aromatic systems due to the positive quadrupole moment; so, the excess anions present in solution in the assemblies studied here may interact with **3** to produce the different self-assembly behavior observed when compared to **1** and **2**.^69,70^ Lastly, the perfluorination of the phenyl ring in gelator **3** also increases both the steric bulk and overall hydrophobicity of the compound compared to **1** and **2**. These properties together must account for the dramatic difference in self-assembly behavior of **3**, but the role of each in the overall supramolecular outcome is unclear without high-resolution packing information.

The assembly environment, determined by the choice of anion used to initiate assembly, also has a considerable impact on the self-assembly of the Fmoc-Phe-DAP derivatives **1–3**. In most cases, it appears that specific anion interactions with the gelators are dominating over any general effects that may be caused by pH differences or Hofmeister series effects. In fact, the only evidence of a potential direct Hofmeister effect for these assemblies is the trend of increasing *G*′ values when gelling **3** with the monovalent salt series. We speculate that the observation of a single fiber morphology type in these samples regardless of the anion identity may allow Hofmeister effects to impact this system, whereas the polymorphism of assemblies of **1** and **2** adds complexity to trends observed for hydrogel formation or viscoelastic moduli of these derivatives. Though the morphological outcomes of assemblies of **1** and **2** could be grouped into four sets by broad similarities, subtle differences within each group were still present, indicating that specific interactions between the gelators and anions are important in the self-assembly process. The absence of a clear Hofmeister effect on the emergent properties of Fmoc-Phe-DAP gelators deviates from the direct Hofmeister effect observed by Ulijn and co-workers for the dipeptide gelator Fmoc-YL, where the viscoelastic moduli of hydrogels formed by Fmoc-YL in the presence of sodium salts decreased across the series from kosmotropes to chaotropes.^56^ However, Fmoc-YL is an anionic gelator that was chosen to directly study Hofmeister effects by ruling out any electrostatic interaction between the gelator and added anions. In the present study of Fmoc-Phe-DAP derivatives, electrostatic interactions between the cationic amine moiety on the gelator and the added anions are central to facilitating the self-assembly of these compounds, and thus, any Hofmeister effects may be overruled by specific anion interactions with the cationic gelators.

Though both the gelator structure and environmental anionic effects clearly impact the self-assembly of these Fmoc-Phe-DAP derivatives, the interplay between these two effects in modulating the emergent material properties observed in this study appears to be idiosyncratic. This is due to our current lack of understanding of the precise nature by which each of these properties influences the overall self-assembly of LMW Fmoc-Phe-derived gelators, since traditional techniques for structural analysis such as crystallography and scattering cannot provide high-resolution structural information about the molecular ordering of these assemblies in their native state. The idiosyncratic observations reported in this work provide significant motivation for continued research into correlating molecular structure and assembly environment to supramolecular outcomes. Structural packing data obtained by emerging technologies such as cryo-EM tomography will be critical in the field of LMW supramolecular assembly if we desire to control the emergent properties of assembly through rational design of gelators.

## Conclusions

Herein, we have demonstrated the effect that salt choice and gelator structure has on the supramolecular assembly of cationic Fmoc-Phe-DAP gelators. Gelators **1–3** each showed a unique set of behaviors when considering the gelation capacity, nanoscale morphology, and emergent viscoelastic properties. Assemblies of gelator **1** could be grouped into three categories based on visual observation and four categories based on morphological patterns, but the viscoelastic moduli for these samples were varied and not obviously correlated to either grouping. Assemblies of **2** consequently showed similar morphological groupings at the nanoscale as were observed for assemblies of **1**, though anion-specific differences were apparent. However, hydrogels of **2** had nearly constant viscoelastic moduli that were independent of the anion choice. Conversely, gelator **3** formed hydrogels composed of the same fiber morphology for all anions examined, but those hydrogels had varied viscoelastic moduli despite the observed similarity of other emergent properties. The apparent idiosyncratic nature of the emergent nanoscale and macroscale properties of these three similar molecules highlights how subtle differences in a supramolecular system can have a large impact on the outcome. Particularly, there exists a complex interplay between how the gelator molecular structure and the self-assembly environment influence self-assembly pathways and the emergent properties of the assembly. These effects must be considered when developing LMW supramolecular gelators for biological applications, since biological fluids are a complex mixture of charged species that may affect self-assembly in unforeseen ways. These findings also underscore the need for high-resolution structural data of the native packing of LMW gelators at the molecular level. This information will be crucial to establish correlations between the chemical structure and emergent supramolecular properties to ultimately allow the rational design of LMW gelators.

## Supporting Information Available

Digital images of assembled samples, TEM images of assemblies, mass spectra, NMR spectra, HPLC spectra, and additional rheological data (PDF)
