# Supplementary material for: Anion Effects on the Supramolecular Self-Assembly of Cationic Phenylalanine Derivatives
Source: Langmuir. 2022 Dec 6;38(50):15494–505. doi: 10.1021/acs.langmuir.2c01394 (PMC9776537; doi:10.1021/acs.langmuir.2c01394)
Supplement: Supplementary file 1 — la2c01394_si_001.pdf [file la2c01394_si_001.pdf]

Supporting Information

Anion Effects on the Supramolecular Self-Assembly of Cationic Phenylalanine Derivatives

*Brittany L. Abraham,[a] Pamela Agredo,[a] Samantha G. Mensah,[a] and Bradley L. Nilsson[a,b]**

[a]Department of Chemistry, University of Rochester, Rochester, NY 14627-0216, USA.

[b] Materials Science Program, University of Rochester, Rochester, NY 14627-0166, USA.

E-mail: bradley.nilsson@rochester.edu

Tel. +1 585 276-3053

SUPPORTING INFORMATION

Contents:

Figure S1. Digital images of assemblies of **1** from 10–15 mM............page S3
Figure S2. Digital images of assemblies of **1** from 1–10 mM............page S4
Figure S3. Digital images of assemblies of **2** from 10–15 mM............page S5
Figure S4. Digital images of assemblies of **2** from 1–10 mM............page S6
Figure S5. Digital images of assemblies of **3** from 1–10 mM............page S7
Figure S6. Digital images of assemblies of **3** from 1–10 mM............page S8
Figure S7. TEM images of assemblies of **1**............page S9
Figure S8. TEM images of assemblies of **1**............page S10
Figure S9. TEM images of assemblies of **2**............page S11
Figure S10. TEM images of assemblies of **2**............page S12
Figure S11. TEM images of assemblies of **3**............page S13
Figure S12. TEM images of assemblies of **3**............page S14
Table S1. TEM nanostructure measurements for hydrogels of **1**............page S15
Table S2. TEM nanostructure measurements for hydrogels of **2**............page S16
Table S3. TEM nanostructure measurements for hydrogels of **3**............page S17
Figure S13. Mass spectrum of **1**............page S18
Figure S14. Mass spectrum of **1** assembled with NaCl............page S19
Figure S15. Mass spectrum of **1** assembled with $NaNO_3$............page S20
Figure S16. Mass spectrum of **1** assembled with $NaClO_4$............page S21
Figure S17. Mass spectrum of **2**............page S22
Figure S18. Mass spectrum of **2** assembled with NaCl............page S23
Figure S19. Mass spectrum of **2** assembled with $NaNO_3$............page S24
Figure S20. Mass spectrum of **2** assembled with $NaClO_4$............page S25
Figure S21. Mass spectrum of **3**............page S26
Figure S22. Mass spectrum of **3** assembled with NaCl............page S27
Figure S23. Mass spectrum of **3** assembled with $NaNO_3$............page S28
Figure S24. Mass spectrum of **3** assembled with $NaClO_4$............page S29
Figure S25. ^{1}H NMR spectra of select assemblies of **1**............page S30
Figure S26. ^{1}H NMR spectra of select assemblies of **2**............page S30
Figure S27. ^{1}H NMR spectra of select assemblies of **3**............page S31
Table S4. Analytical HPLC method conditions............page S31

Figure S28. HPLC traces of select assemblies of **1**....page S32
Figure S29. HPLC traces of select assemblies of **2**....page S32
Figure S30. HPLC traces of select assemblies of **3**....page S33
Figure S31. Strain sweep data for hydrogels of **1**....page S33
Figure S32. Strain sweep data for hydrogels of **2**....page S34
Figure S33. Strain sweep data for hydrogels of **3**....page S35
Figure S34. Frequency sweep data for hydrogels of **1**....page S36
Figure S35. Frequency sweep data for hydrogels of **2**....page S37
Figure S36. Frequency sweep data for hydrogels of **3**....page S38
Table S5. Average storage (G′) and loss (G″) moduli of hydrogels of **1–3**....page S39
Table S6. pH values of assemblies of **1–3**....page S40
Figure S37. ^{1}H NMR spectra of **1** to determine monomer concentration....page S41
Figure S38. ^{1}H NMR spectra of **2** to determine monomer concentration....page S42
Figure S39. ^{1}H NMR spectra of **3** to determine monomer concentration....page S43
References....page S43

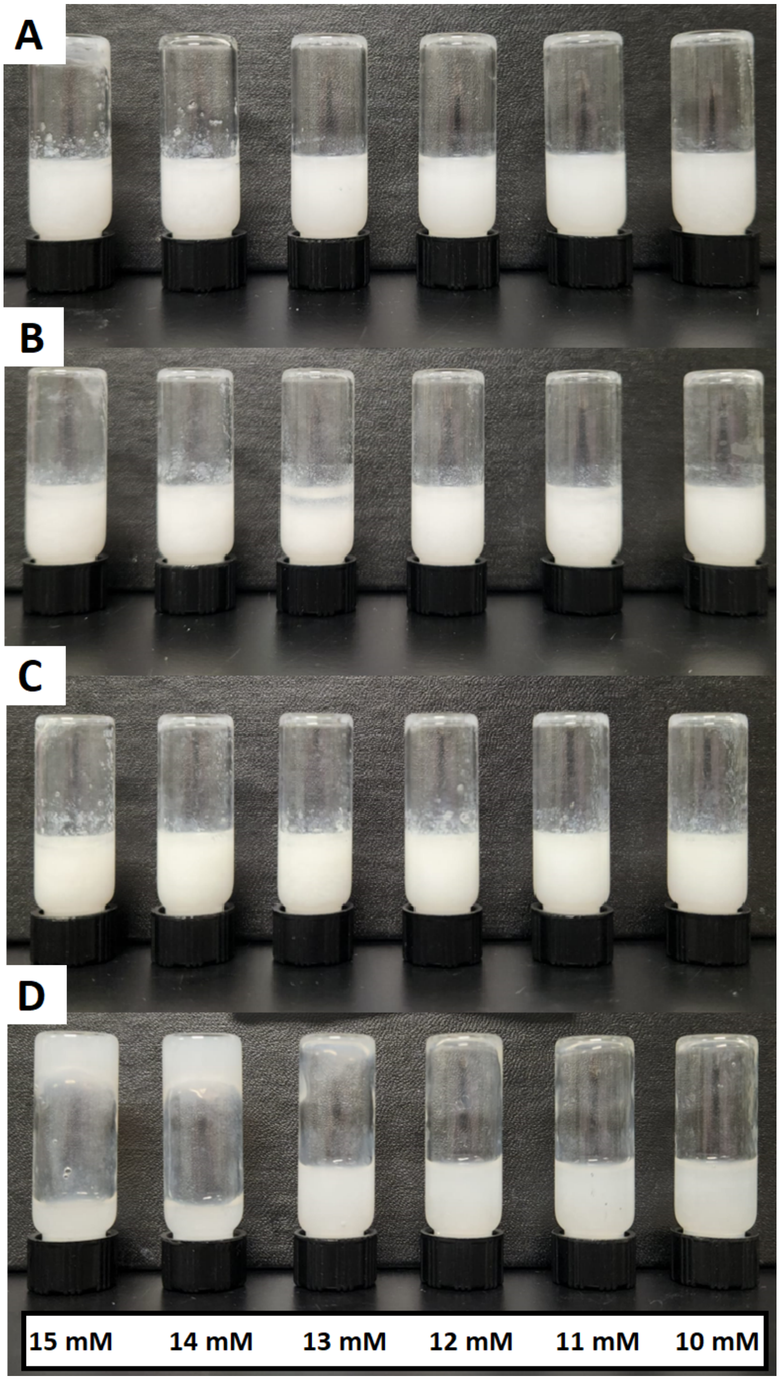

Figure S1. Digital images of Fmoc-Phe-DAP (**1**) assemblies prepared to determine critical gelation concentration. Assemblies were formed between 10 mM–15 mM gelator concentration by adding 100 mM of (A) $Na_3C_6H_5O_7$ (B) Na_2SO_4 (C) Na_2HPO_4 (D) NaSCN.

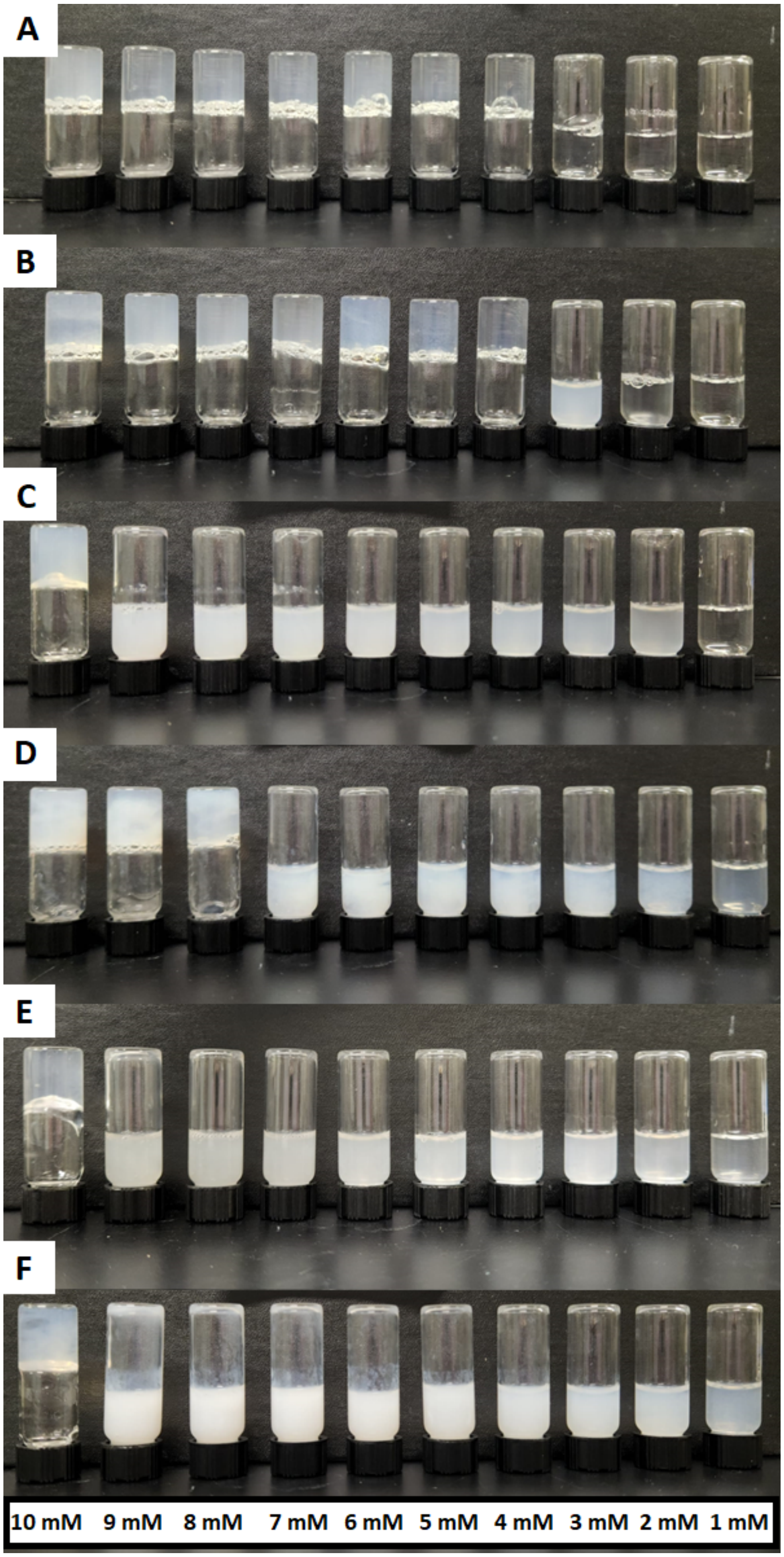

Figure S2. Digital images of Fmoc-Phe-DAP (**1**) assemblies prepared to determine critical gelation concentration. Assemblies were formed between 1 mM–10 mM gelator concentration by adding 100 mM of (A) NaOAc (B) NaCl (C) NaBr (D) $NaNO_3$ (E) NaI (F) $NaClO_4$.

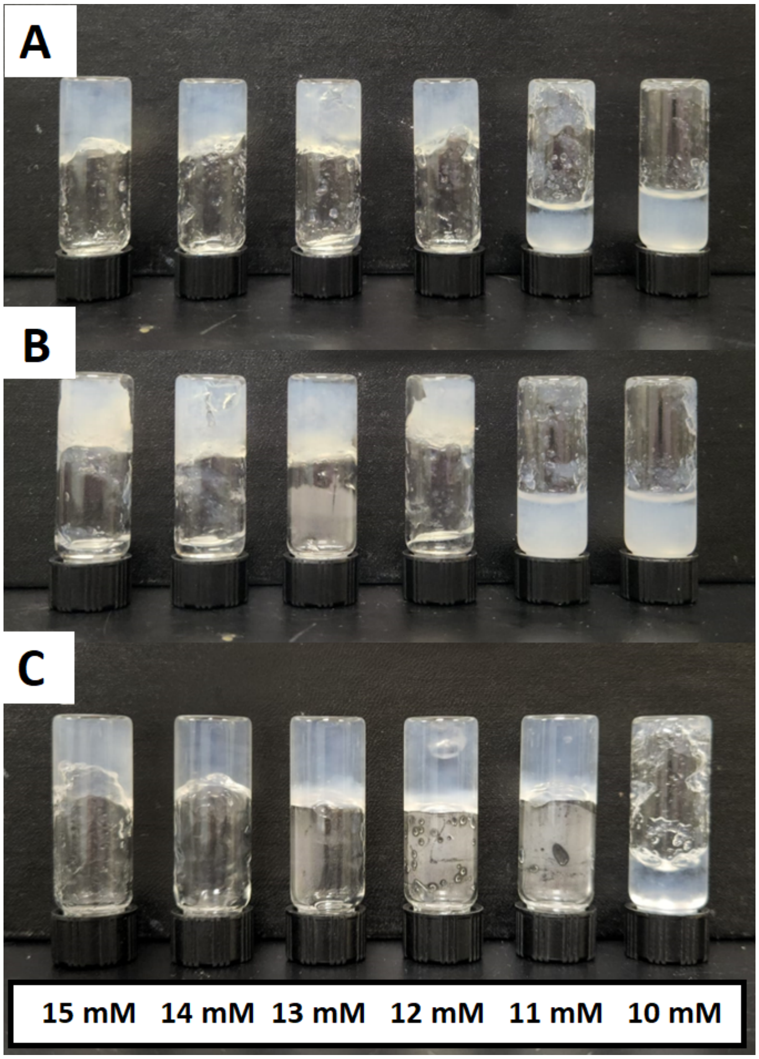

Figure S3. Digital images of Fmoc-Phe-DAP (**1**) assemblies prepared to determine critical gelation concentration. Assemblies were formed between 10 mM–15 mM gelator concentration by adding 100 mM of (A) Na_2SO_4 (B) Na_2HPO_4 (C) $NaClO_4$.

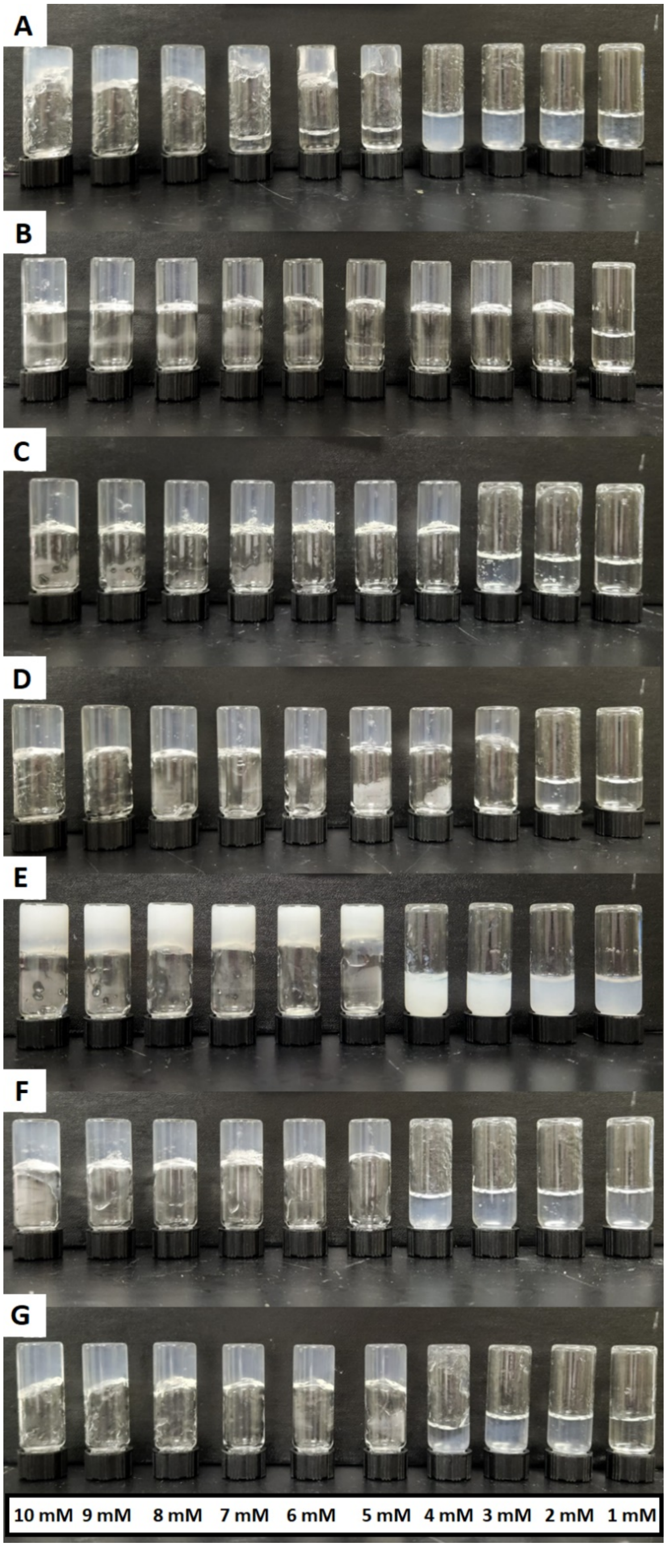

Figure S4. Digital images of Fmoc-Phe-DAP (**1**) assemblies prepared to determine critical gelation concentration. Assemblies were formed between 1 mM–10 mM gelator concentration by adding 100 mM of (A) $Na_3C_6H_5O_7$ (B) NaOAc (C) NaCl (D) NaBr (E) $NaNO_3$ (F) NaI (G) NaSCN.

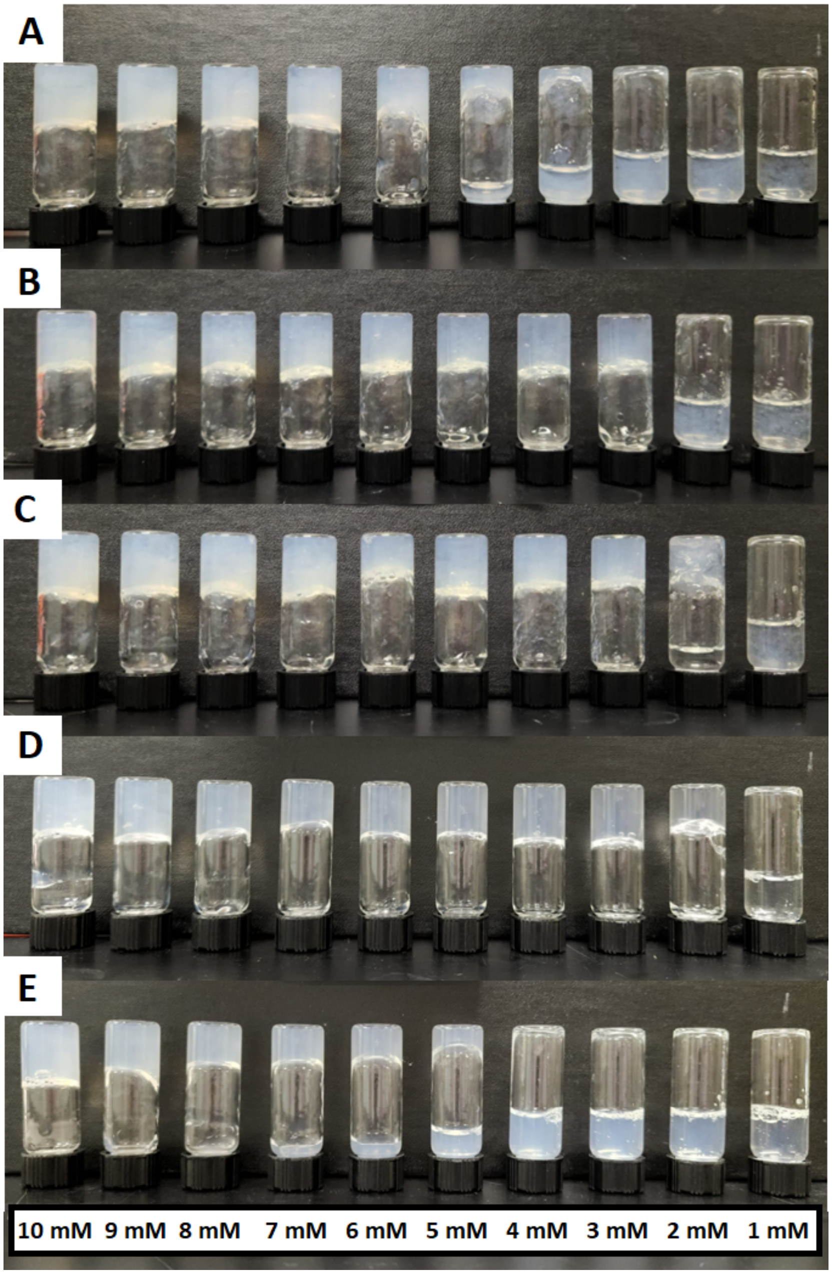

Figure S5. Digital images of Fmoc-Phe-DAP (**1**) assemblies prepared to determine critical gelation concentration. Assemblies were formed between 10 mM–15 mM gelator concentration by adding 100 mM of (A) $Na_3C_6H_5O_7$ (B) Na_2SO_4 (C) Na_2HPO_4 (D) NaOAc (E) NaCl.

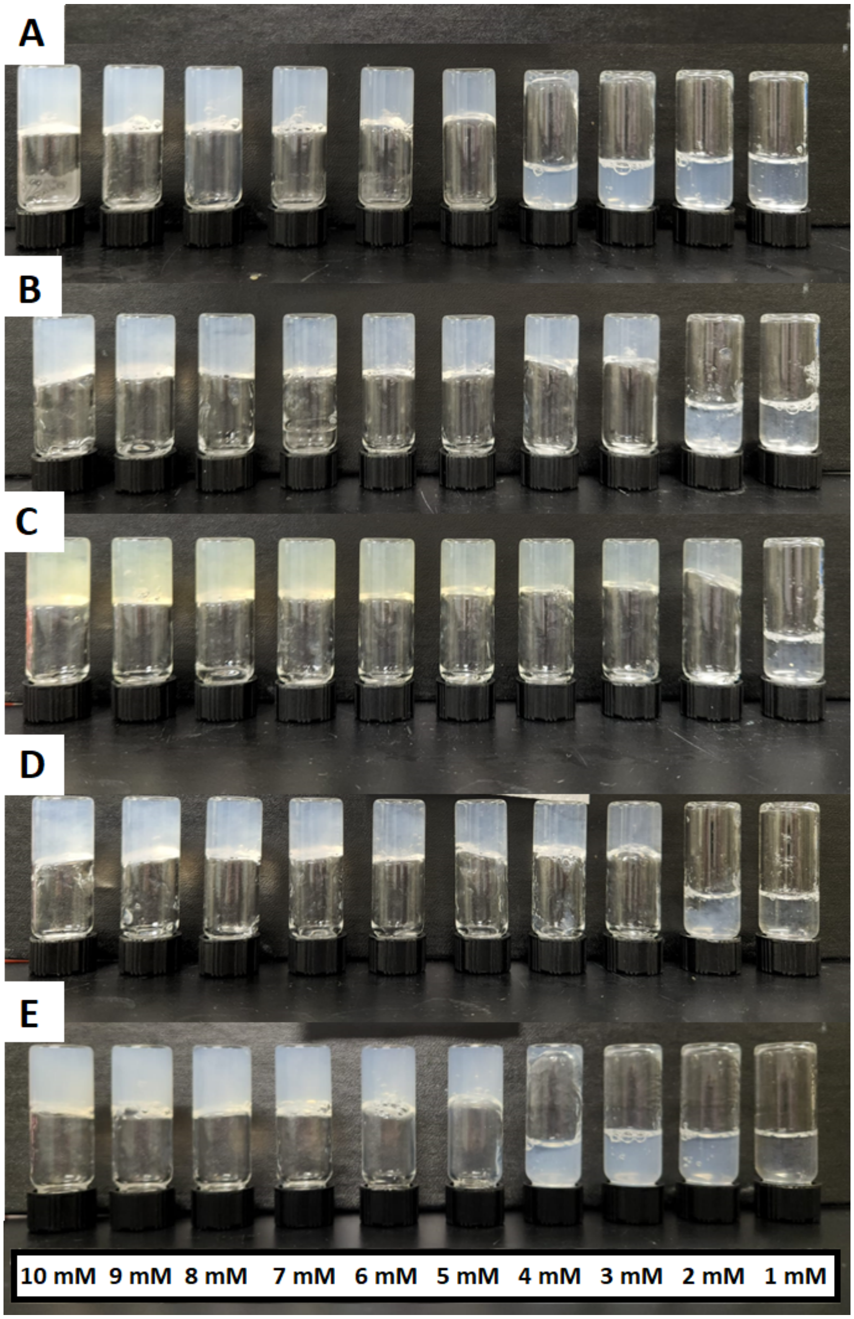

Figure S6. Digital images of Fmoc-Phe-DAP (**1**) assemblies prepared to determine critical gelation concentration. Assemblies were formed between 10 mM–15 mM gelator concentration by adding 100 mM of (A) NaBr (B) $NaNO_3$ (C) NaI (D) $NaClO_4$ (E) NaSCN.

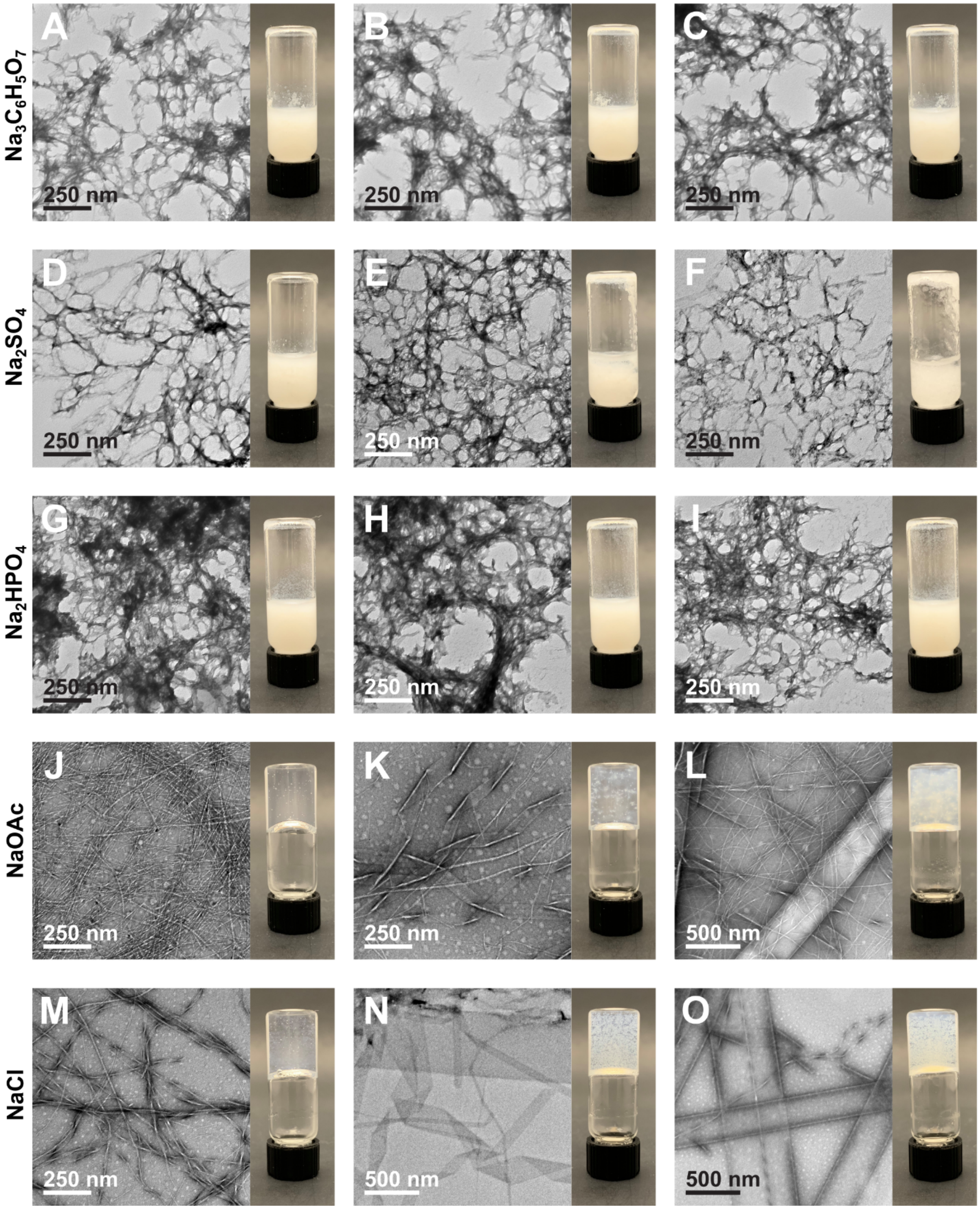

Figure S1. TEM and digital images of Fmoc-Phe-DAP (**1**) assemblies taken 30 minutes, 24 hours, and 1 week after triggering gelation using various salts. (**A–C**) $Na_3C_6H_5O_7$ assembly after (A) 30 minutes, (B) 24 hours, and (C) 1 week. (**D–F**) Na_2SO_4 assembly after (D) 30 minutes, (E) 24 hours, and (F) 1 week. (**G–I**) Na_2HPO_4 assemblies after (G) 30 minutes, (H) 24 hours, and (I) 1 week. (**J–L**) NaOAc hydrogels after (J) 30 minutes, (K) 24 hours, and (L) 1 week. (**M–O**) NaCl hydrogels after (M) 30 minutes, (N) 24 hours, and (O) 1 week.

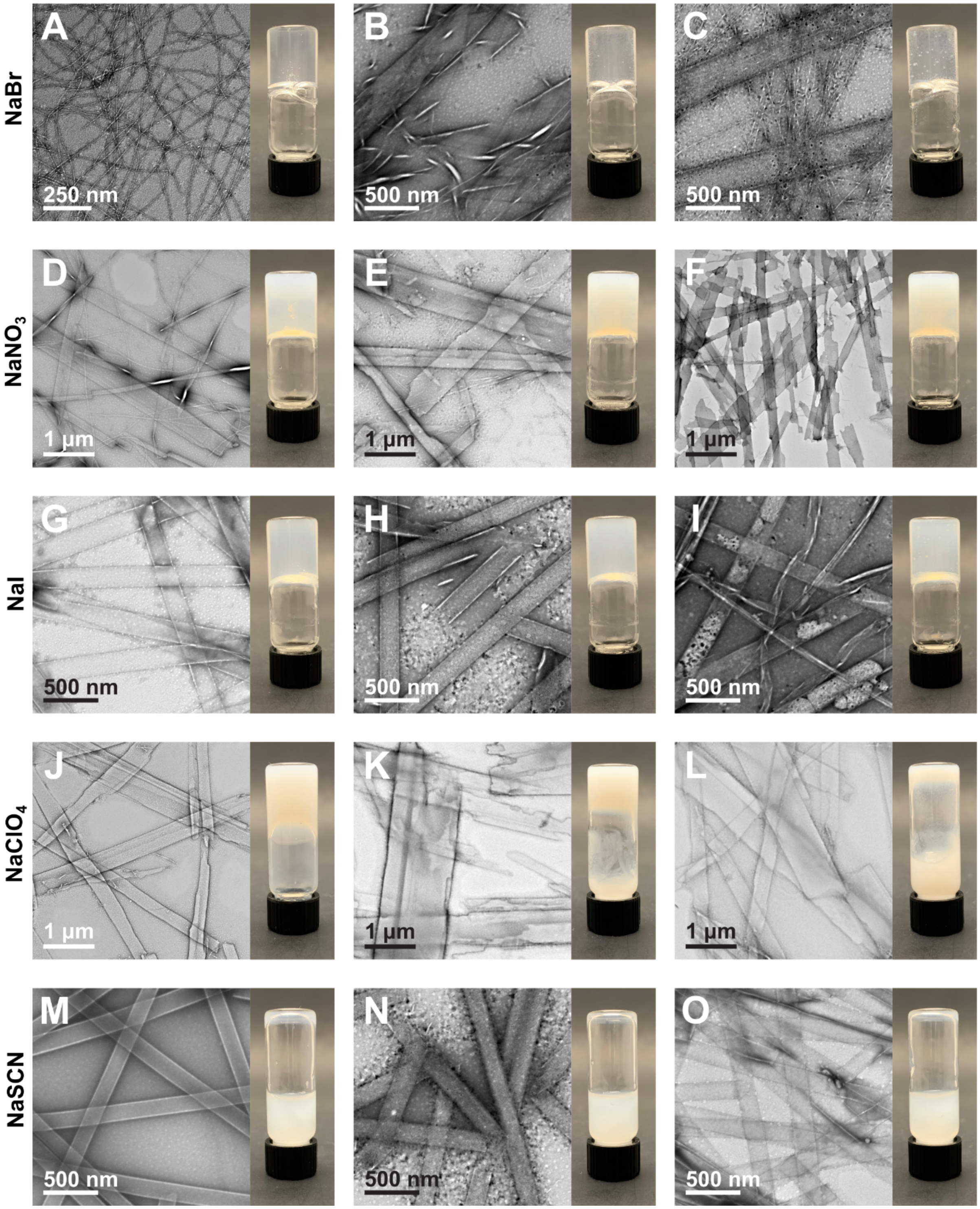

Figure S2. TEM and digital images of Fmoc-Phe-DAP (**1**) assemblies taken 30 minutes, 24 hours, and 1 week after triggering gelation using various salts. (**A–C**) NaBr hydrogel after (A) 30 minutes, (B) 24 hours, and (C) 1 week. (**D–F**) $NaNO_3$ hydrogel after (D) 30 minutes, (E) 24 hours, and (F) 1 week. (**G–I**) NaI hydrogel after (G) 30 minutes, (H) 24 hours, and (I) 1 week. (**J–L**) $NaClO_4$ hydrogel after (J) 30 minutes, (K) 24 hours, and (L) 1 week. (**M–O**) NaSCN hydrogels after (M) 30 minutes, (N) 24 hours, and (O) 1 week.

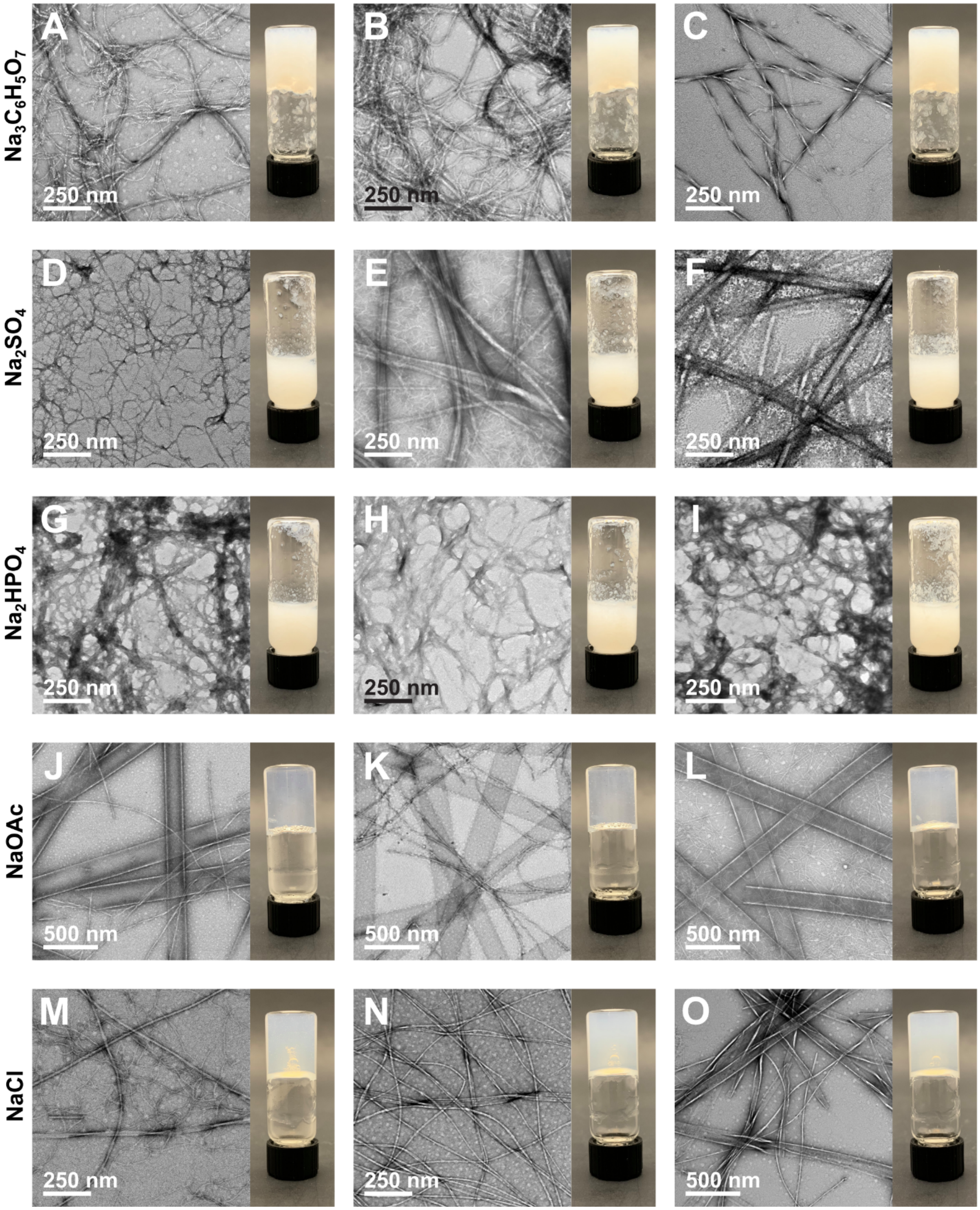

Figure S3. TEM and digital images of Fmoc-3F-Phe-DAP (**2**) assemblies taken 30 minutes, 24 hours, and 1 week after triggering gelation using various salts. (**A–C**) $Na_3C_6H_5O_7$ hydrogel after (A) 30 minutes, (B) 24 hours, and (C) 1 week. (**D–F**) Na_2SO_4 assemblies after (D) 30 minutes, (E) 24 hours, and (F) 1 week. (**G–I**) Na_2HPO_4 assemblies after (G) 30 minutes, (H) 24 hours, and (I) 1 week. (**J–L**) NaOAc hydrogel after (J) 30 minutes, (K) 24 hours, and (L) 1 week. (**M–O**) NaCl hydrogel after (M) 30 minutes, (N) 24 hours, and (O) 1 week.

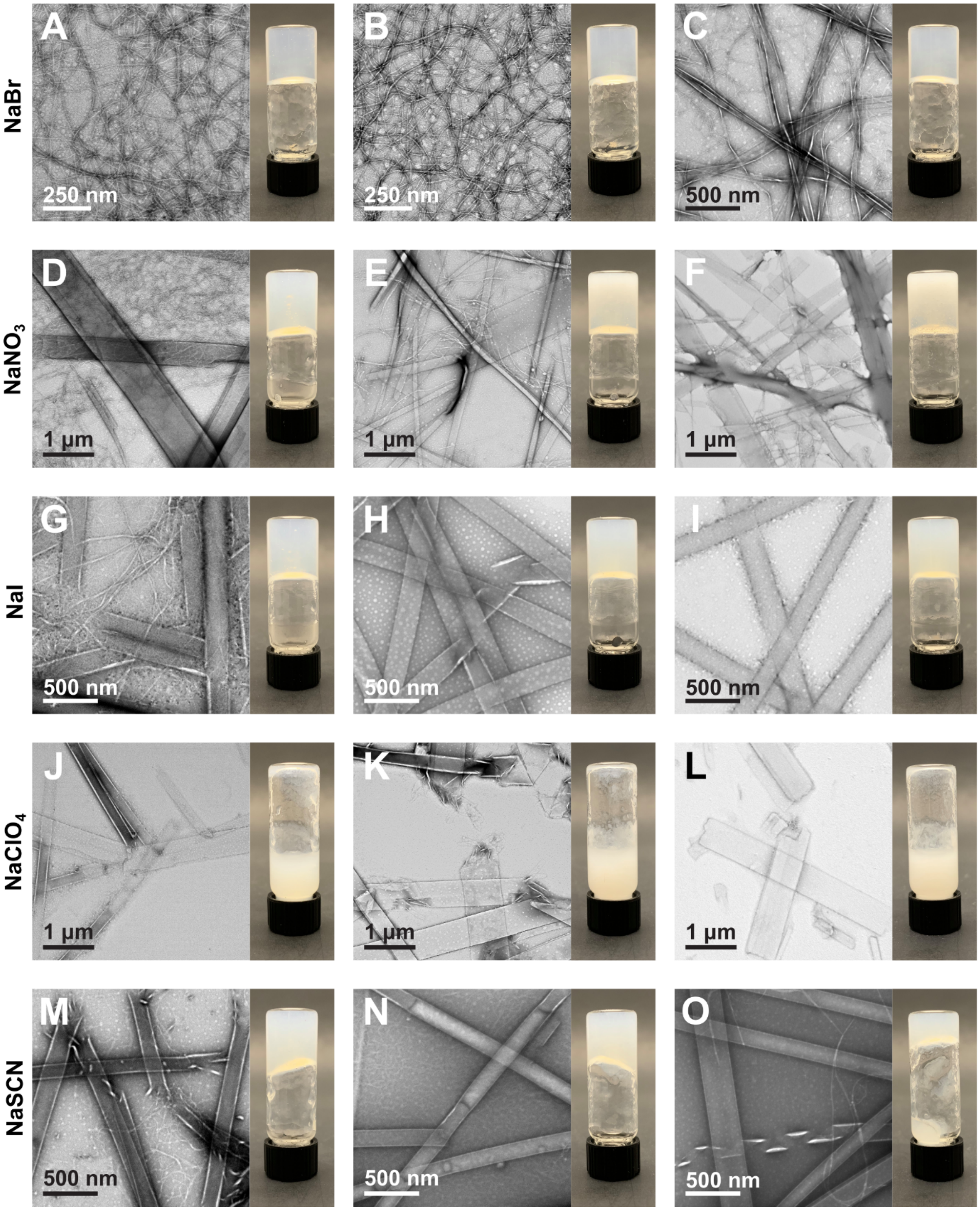

Figure S4. TEM and digital images of Fmoc-3F-Phe-DAP (**2**) assemblies taken 30 minutes, 24 hours, and 1 week after triggering gelation using various salts. (**A–C**) NaBr hydrogel after (A) 30 minutes, (B) 24 hours, and (C) 1 week. (**D–F**) $NaNO_3$ hydrogel after (D) 30 minutes, (E) 24 hours, and (F) 1 week. (G–I) NaI hydrogel after (G) 30 minutes, (H) 24 hours, and (I) 1 week. (**J–L**) $NaClO_4$ assemblies after (J) 30 minutes, (K) 24 hours, and (L) 1 week. (**M–O**) NaSCN hydrogel after (M) 30 minutes, (N) 24 hours, and (O) 1 week.

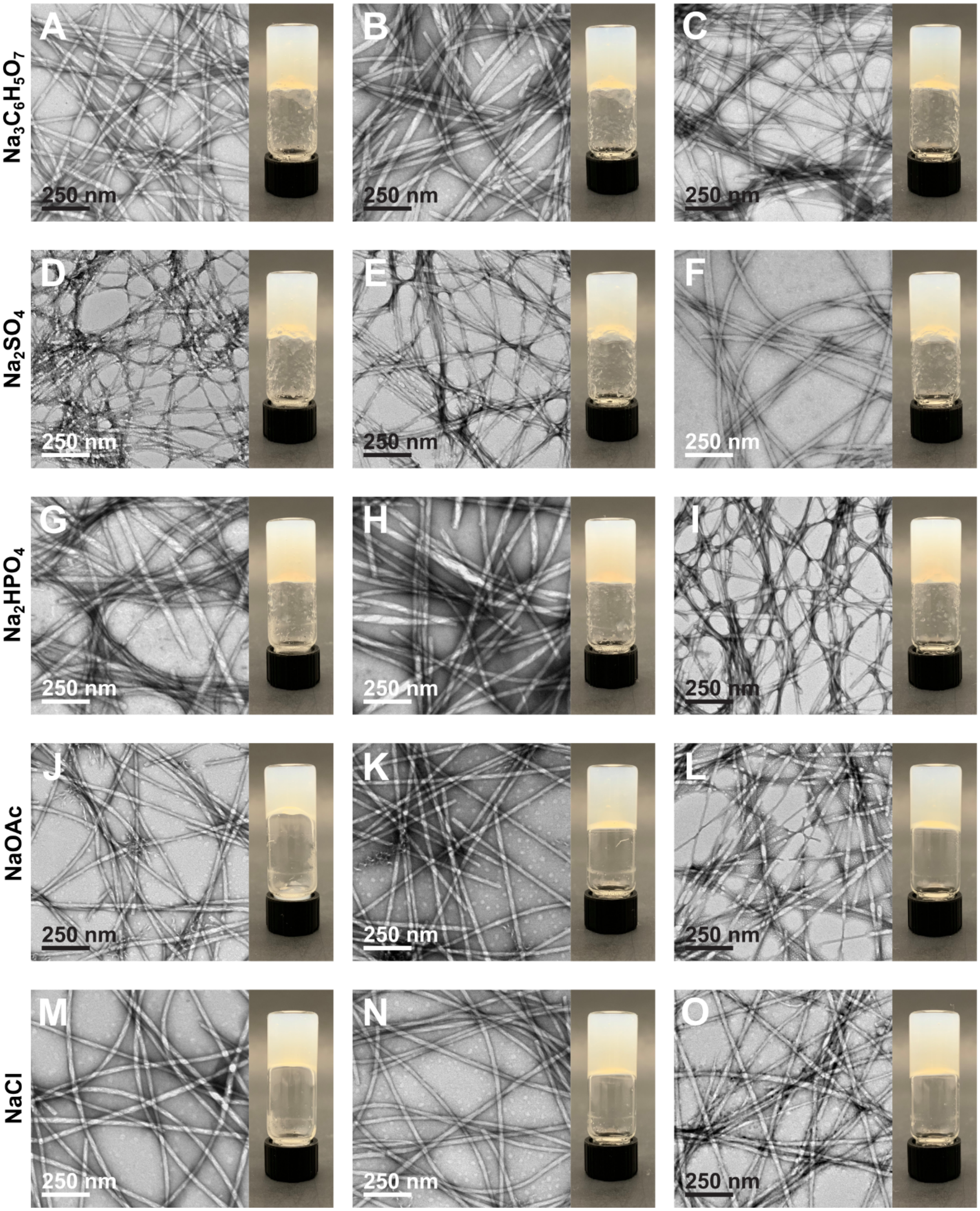

Figure S5. TEM and digital images of hydrogels of Fmoc-F_5-Phe-DAP (**3**) taken 30 minutes, 24 hours, and 1 week after triggering gelation using various salts. (**A–C**) $Na_3C_6H_5O_7$ hydrogel after (A) 30 minutes, (B) 24 hours, and (C) 1 week. (**D–F**) Na_2SO_4 hydrogel after (D) 30 minutes, (E) 24 hours, and (F) 1 week. (**G–I**) Na_2HPO_4 hydrogel after (G) 30 minutes, (H) 24 hours, and (I) 1 week. (**J–L**) NaOAc hydrogel after (J) 30 minutes, (K) 24 hours, and (L) 1 week. (**M–O**) NaCl hydrogel after (M) 30 minutes, (N) 24 hours, and (O) 1 week.

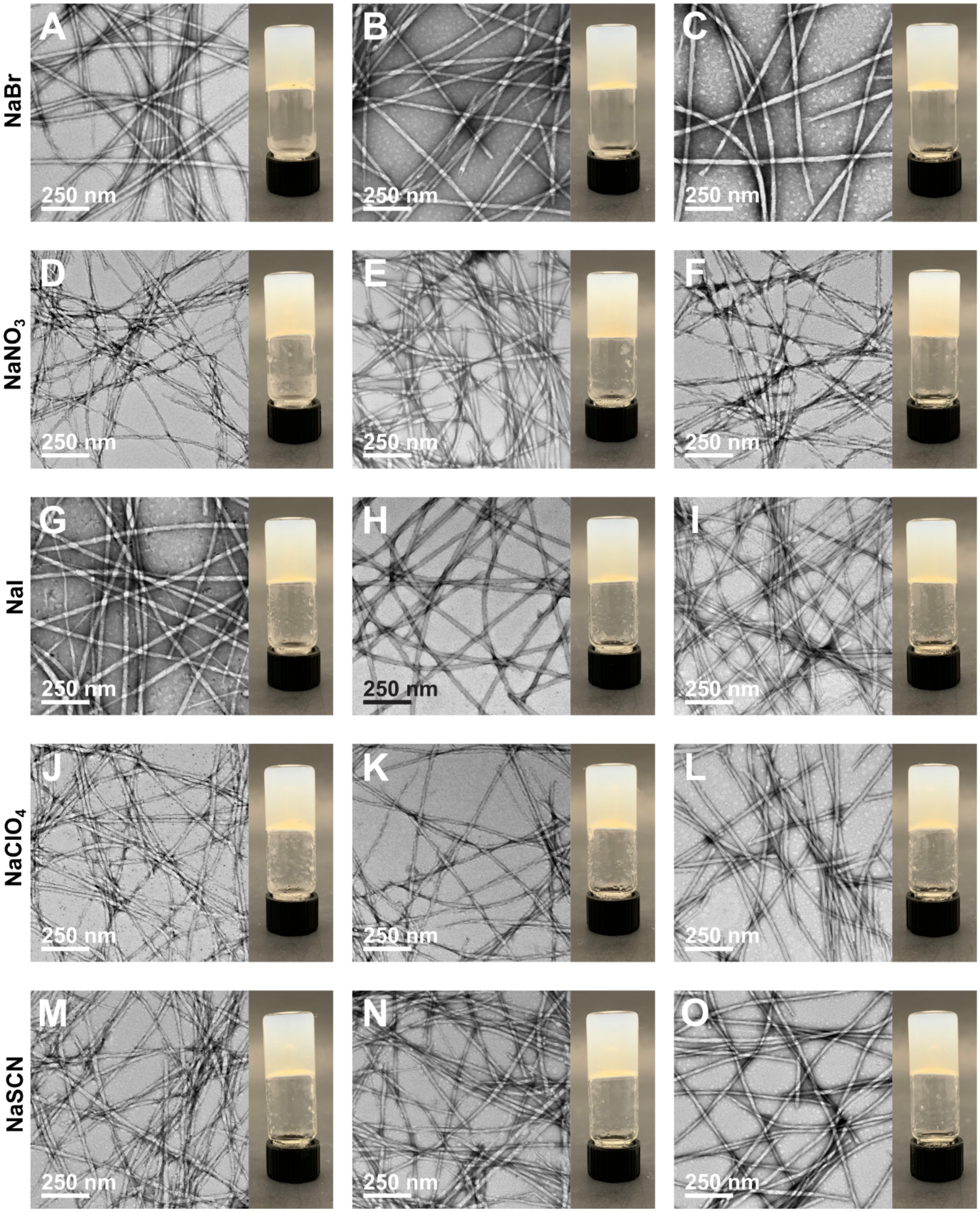

Figure S6. TEM and digital images of hydrogels of Fmoc-F_5-Phe-DAP (**3**) taken 30 minutes, 24 hours, and 1 week after triggering gelation using various salts. (**A–C**) NaBr hydrogel after (A) 30 minutes, (B) 24 hours, and (C) 1 week. (**D–F**) $NaNO_3$ hydrogel after (D) 30 minutes, (E) 24 hours, and (F) 1 week. (**G–I**) NaI hydrogel after (G) 30 minutes, (H) 24 hours, and (I) 1 week. (**J–L**) $NaClO_4$ hydrogel after (J) 30 minutes, (K) 24 hours, and (L) 1 week. (**M–O**) NaSCN hydrogel after (M) 30 minutes, (N) 24 hours, and (O) 1 week.

Table S1. Measurements of the width of nanostructures in hydrogels of Fmoc-Phe-DAP (**1**). Error is reported as the standard deviation about the mean of at least 100 measurements. A range is provided when there was a large variance in nanostructure size or relatively few structures.

	**30 minutes**		**1 week**	
**Salt**	**Morphology**	**Width (nm)**	**Morphology**	**Width (nm)**
$Na_3C_6H_5O_7$	Amorphous precipitate	N/A	Amorphous precipitate	N/A
Na_2SO_4	Amorphous precipitate	N/A	Amorphous precipitate	N/A
Na_2HPO_4	Amorphous precipitate	N/A	Amorphous precipitate	N/A
NaOAc	Fibrils	5.7 ± 0.8	Fibrils<br>Nanoribbons<br>Nanotubes	6.6 ± 1.1<br>12.0–232.0<br>252.6–497.4
NaCl	Fibrils<br>Nanoribbons	7.9 ± 1.2<br>6.1–51.7	Nanoribbons<br>Nanotubes	23.2–465.4<br>227.0–600.6
NaBr	Fibrils	6.4 ± 0.7	Fibrils<br>Nanoribbons<br>Nanotubes	9.5 ± 1.5<br>17.0–177.7<br>187.4–342.0
$NaNO_3$	Fibrils<br>Nanosheets	10.7 ± 1.2<br>37.8–486.4	Fibrils<br>Nanosheets	11.1 ± 1.9<br>76.2–803.5
NaI	Nanotubes	210.1 ± 22.6	Nanotubes	200.0 ± 20.0
$NaClO_4$	Nanosheets	87.6–2222.8	Nanosheets	113.9–2887.8
NaSCN	Nanotubes	165.4 ± 19.6	Nanotubes	159.1 ± 12.6

Table S2. Measurements of the width of nanostructures in hydrogels Fmoc-3F-Phe-DAP (**2**). Error is reported as the standard deviation about the mean of at least 100 measurements. A range is provided when there was a large variance in nanostructure size or relatively few structures.

	**30 minutes**		**1 week**	
**Salt**	**Morphology**	**Width (nm)**	**Morphology**	**Width (nm)**
$Na_3C_6H_5O_7$	Fibrils	14.5 ± 3.5	Twisted fibers	27.0 ± 5.7
Na_2SO_4	Fibrils	9.4 ± 1.3	Twisted fibers	27.6 ± 5.1
Na_2HPO_4	Amorphous precipitate	N/A	Amorphous precipitate	N/A
NaOAc	Fibrils<br>Nanotubes	11.1 ± 1.6<br>198.7 ± 24.6	Fibrils<br>Nanotubes	12.3 ± 2.2<br>195.7 ± 23.7
NaCl	Fibrils<br>Bundled fibers	6.1 ± 0.7<br>13.6 ± 1.9	Nanoribbons<br>Nanotubes	78.2 ± 20.7<br>67.2–81.3
NaBr	Fibrils	5.5 ± 0.9	Fibrils<br>Nanoribbons<br>Nanotubes	6.4 ± 0.8<br>75.9–128.8<br>56.4–109.1
$NaNO_3$	Fibrils<br>Nanosheets	6.3 ± 0.9<br>111.5–1018.0	Fibrils<br>Nanosheets	16.8 ± 2.2<br>125.9–1462.3
NaI	Fibrils<br>Nanotubes	17.0 ± 4.2<br>194.1 ± 17.9	Nanotubes	200.3 ± 18.1
$NaClO_4$	Nanotubes<br>Nanosheets	202.7 ± 15.0<br>44.5–1038.8	Nanosheets	65.4–2689.9
NaSCN	Nanotubes	189.8 ± 26.7	Nanotubes	155.8 ± 19.1

Table S3. Measurements of the width of fibers in hydrogels of Fmoc-F_5-Phe-DAP (**3**) from TEM images taken 30 minutes after assembly (samples remained unchanged after 1 week). Error is reported as the standard deviation about the mean of at least 100 measurements.

Salt	Morphology	Width (nm)
$Na_3C_6H_5O_7$	Nanofibers	21.9 ± 2.3
Na_2SO_4	Nanofibers	20.4 ± 2.4
Na_2HPO_4	Nanofibers	28.7 ± 4.5
NaOAc	Nanofibers	22.7 ± 2.8
NaCl	Nanofibers	24.1 ± 2.8
NaBr	Nanofibers	23.1 ± 3.0
$NaNO_3$	Nanofibers	21.9 ± 3.3
NaI	Nanofibers	25.1 ± 3.0
$NaClO_4$	Nanofibers	21.8 ± 2.7
NaSCN	Nanofibers	21.2 ± 2.6

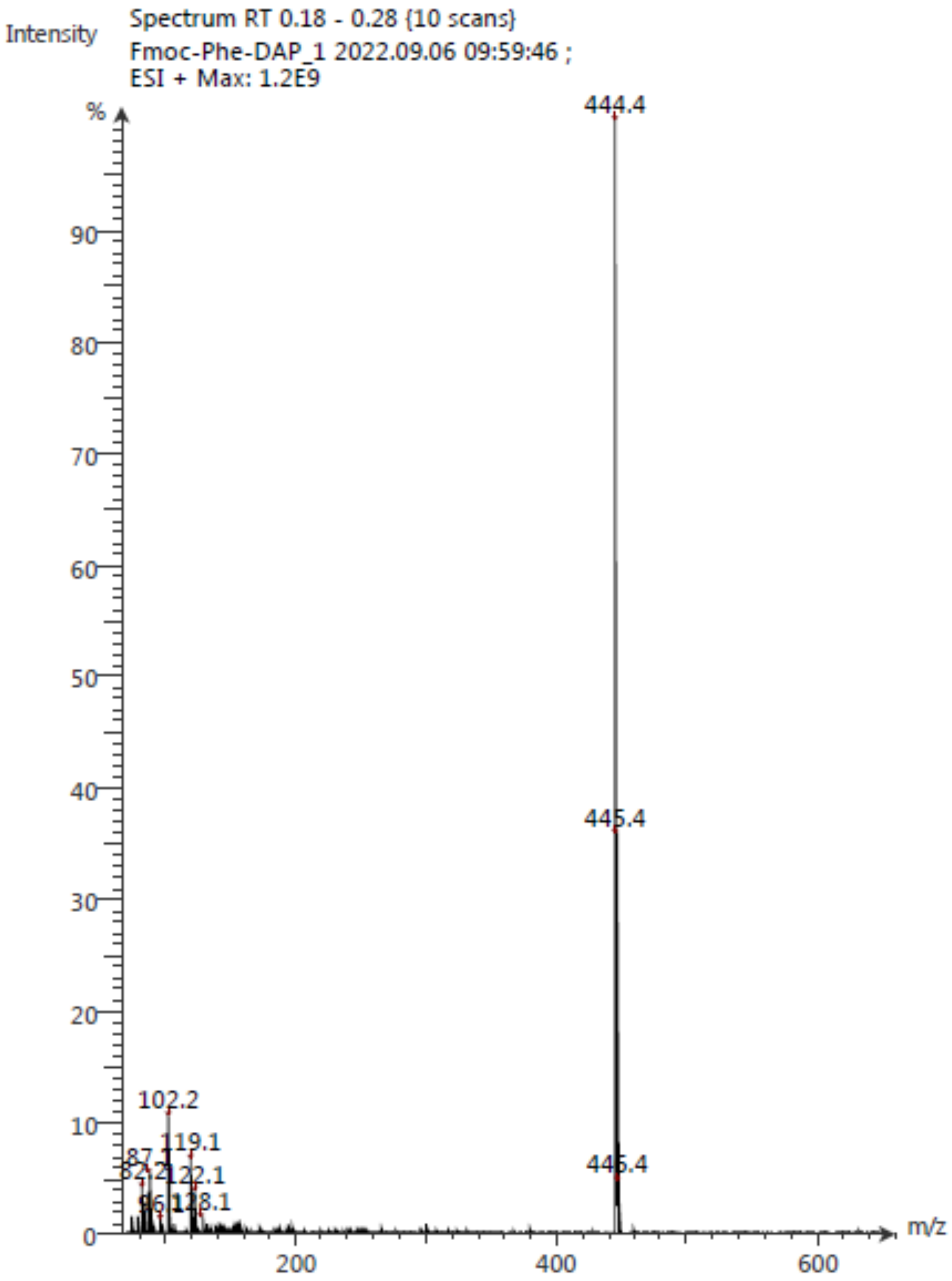

Figure S13. Mass spectrum of Fmoc-Phe-DAP (**1**).

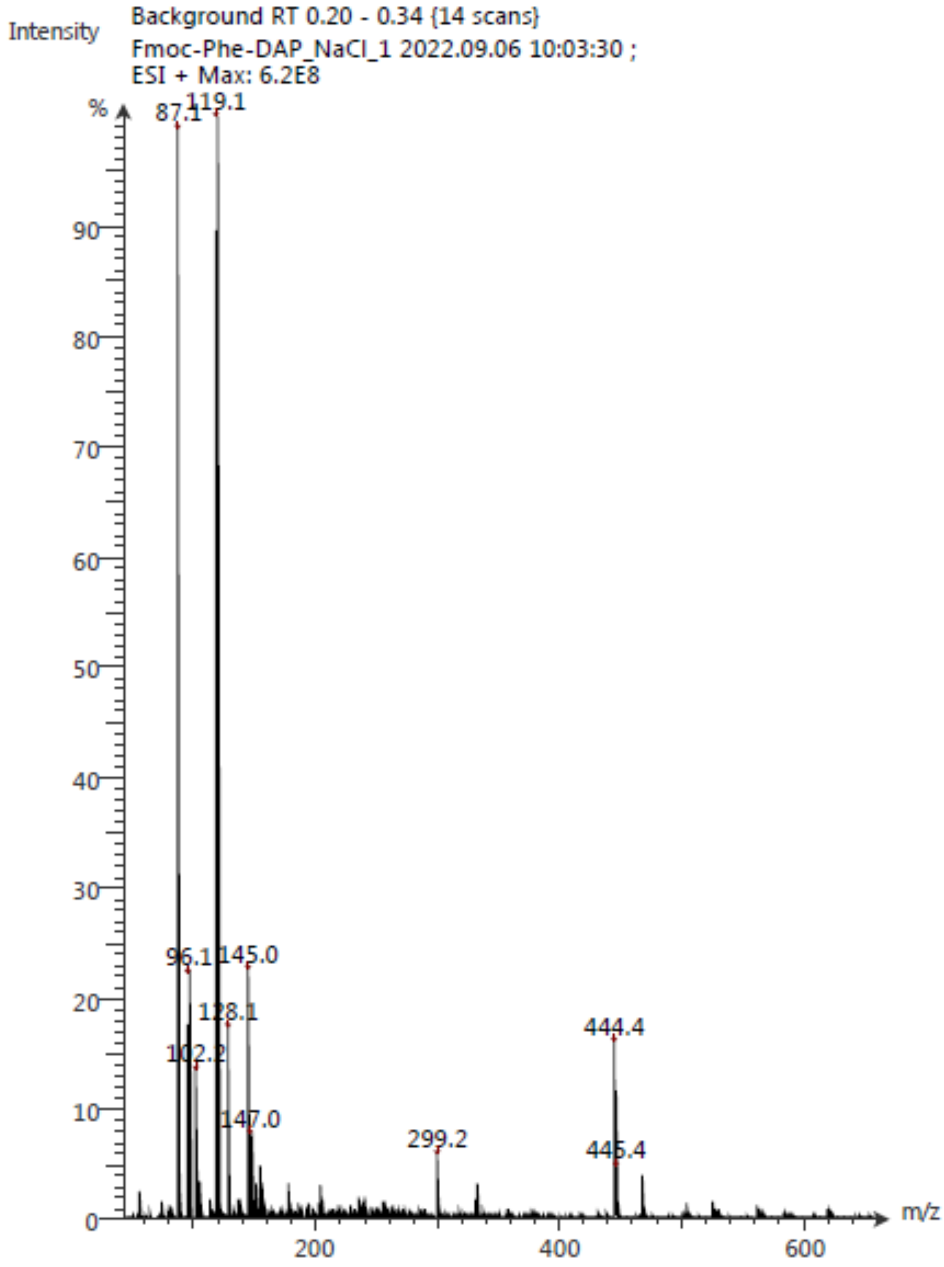

Figure S14. Mass spectrum of Fmoc-Phe-DAP (**1**) after 1 week of gelation with 100 mM NaCl.

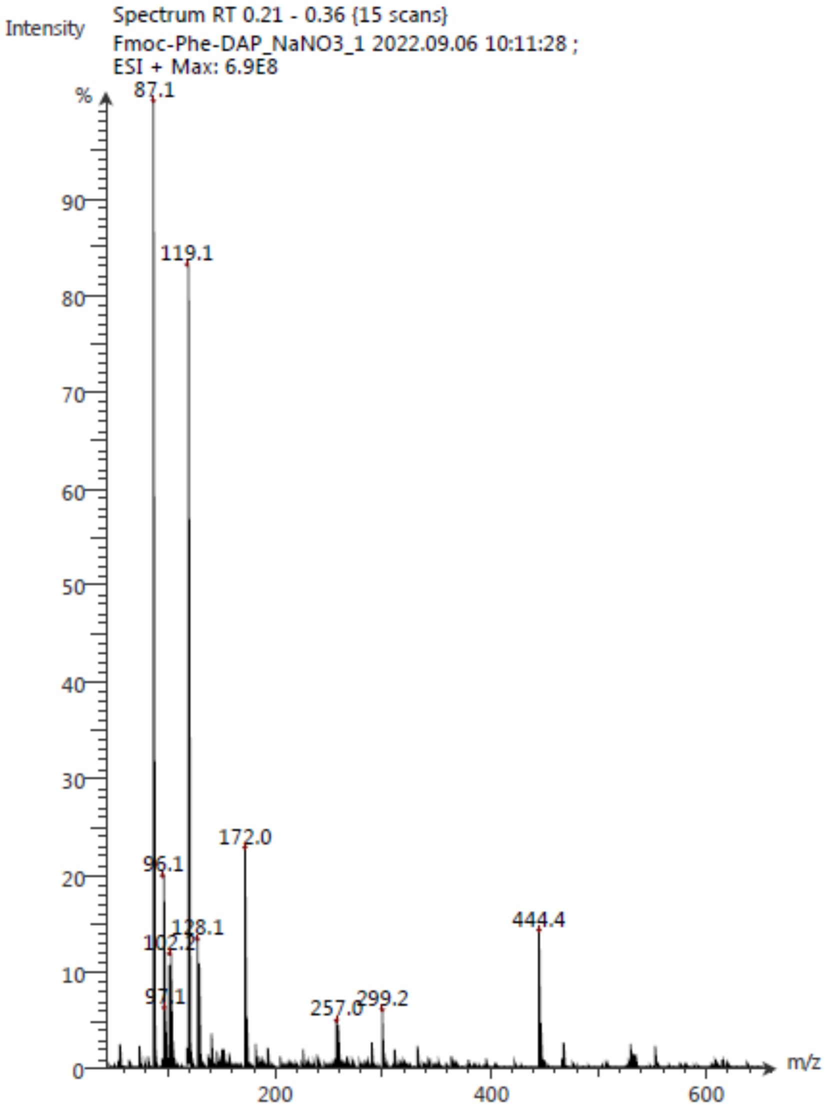

Figure S15. Mass spectrum of Fmoc-Phe-DAP (**1**) after 1 week of gelation with 100 mM $NaNO_3$.

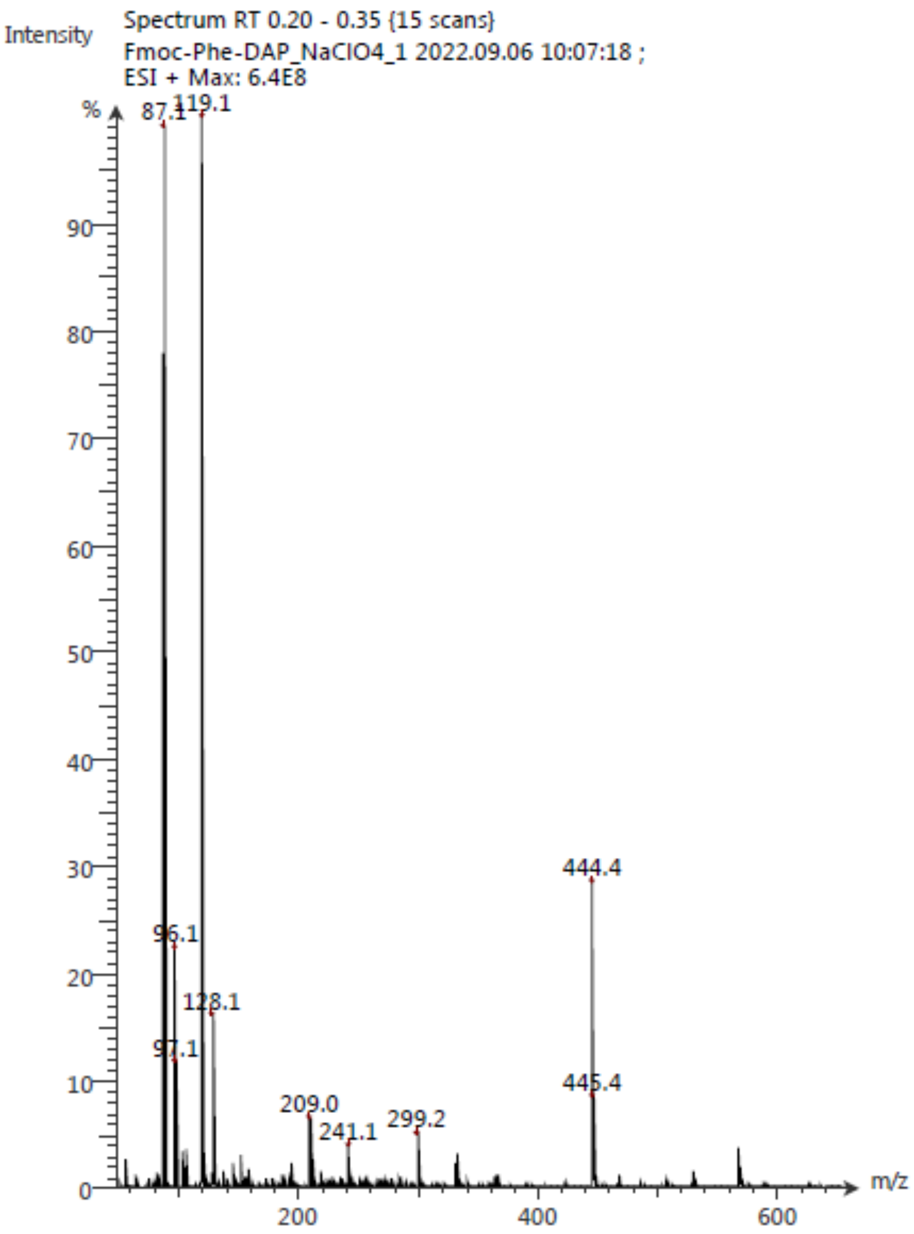

Figure S16. Mass spectrum of Fmoc-Phe-DAP (**1**) after 1 week of gelation with 100 mM $NaClO_4$.

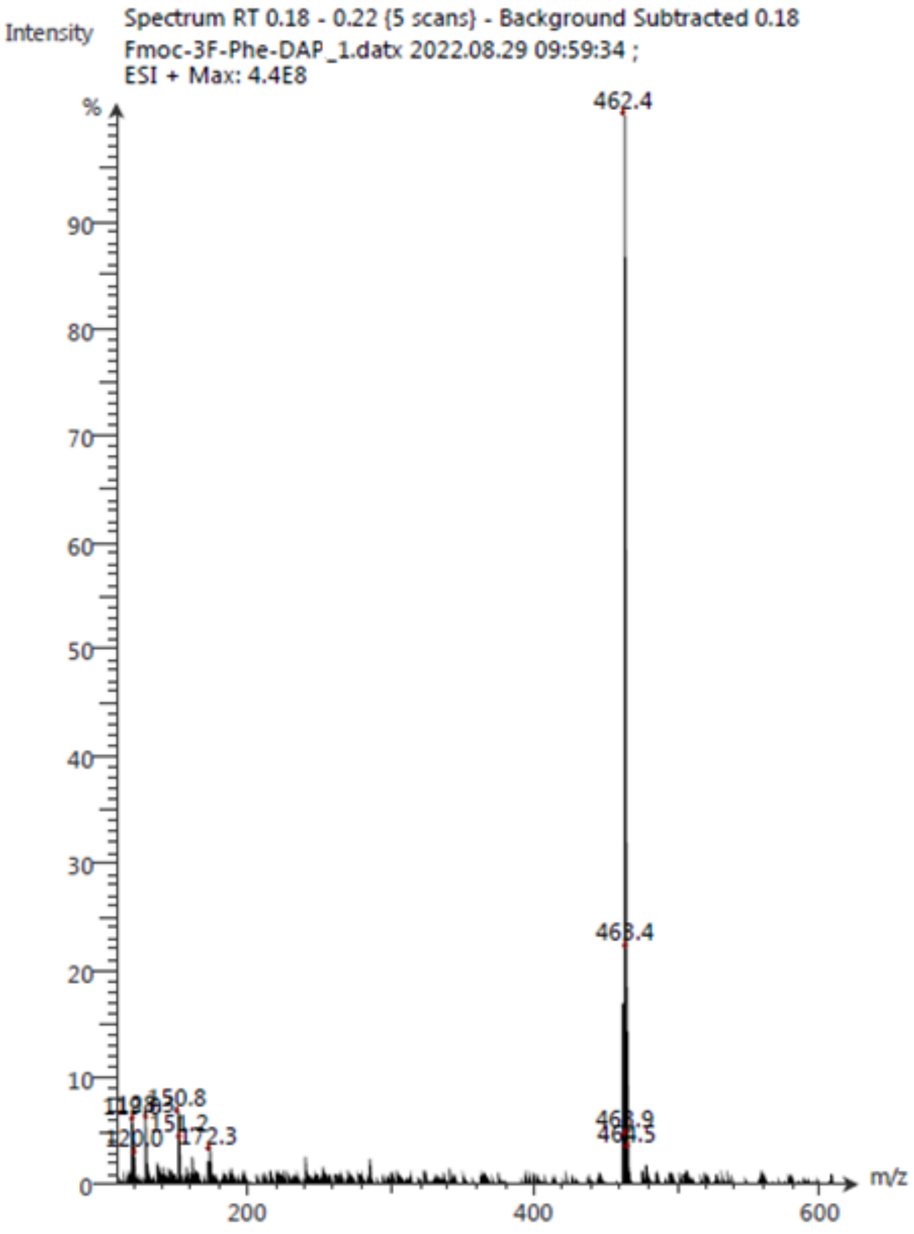

Figure S17. Mass spectrum of Fmoc-3F-Phe-DAP (**2**).

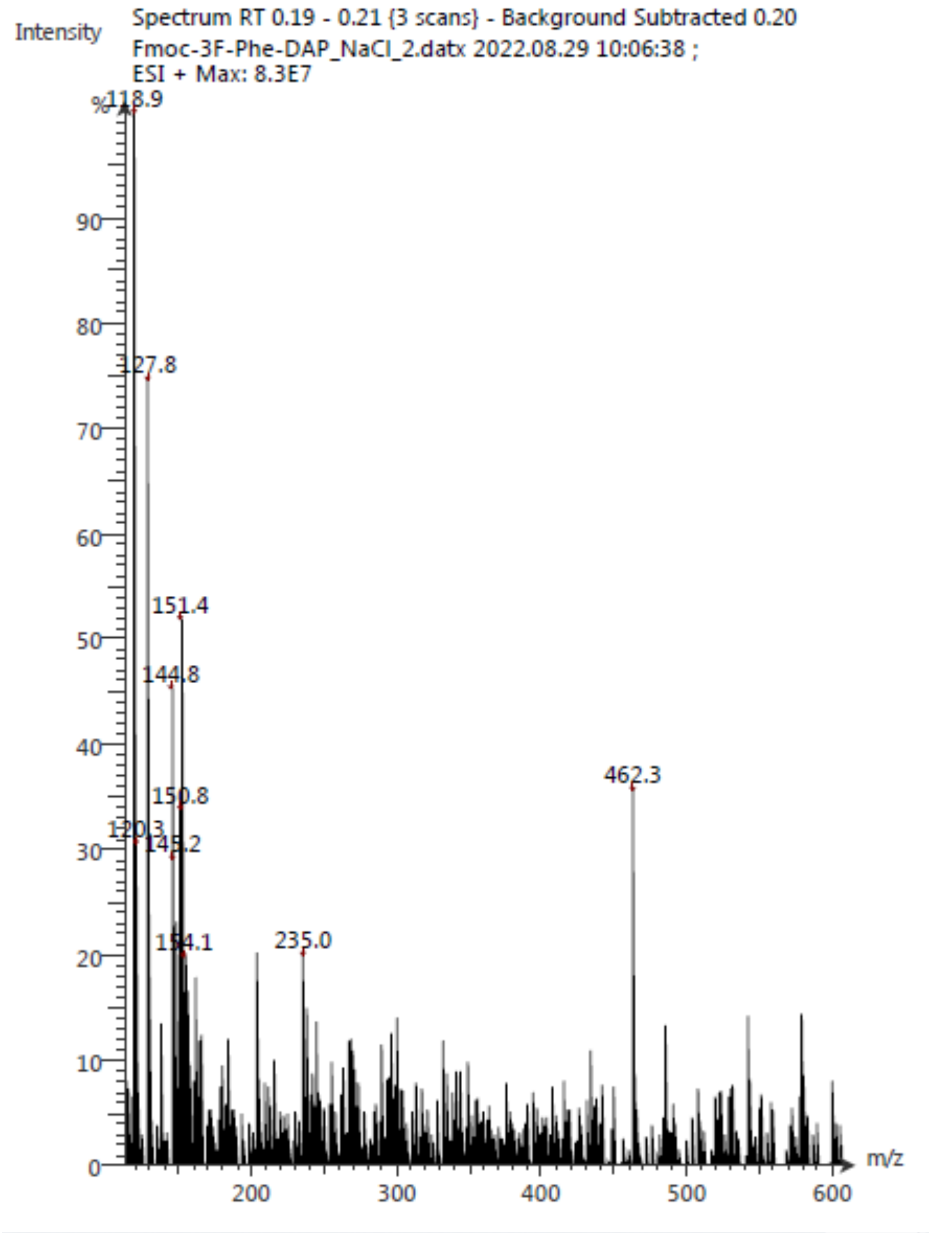

Figure S18. Mass spectrum of Fmoc-3F-Phe-DAP (**2**) after 1 week of gelation with 100 mM NaCl.

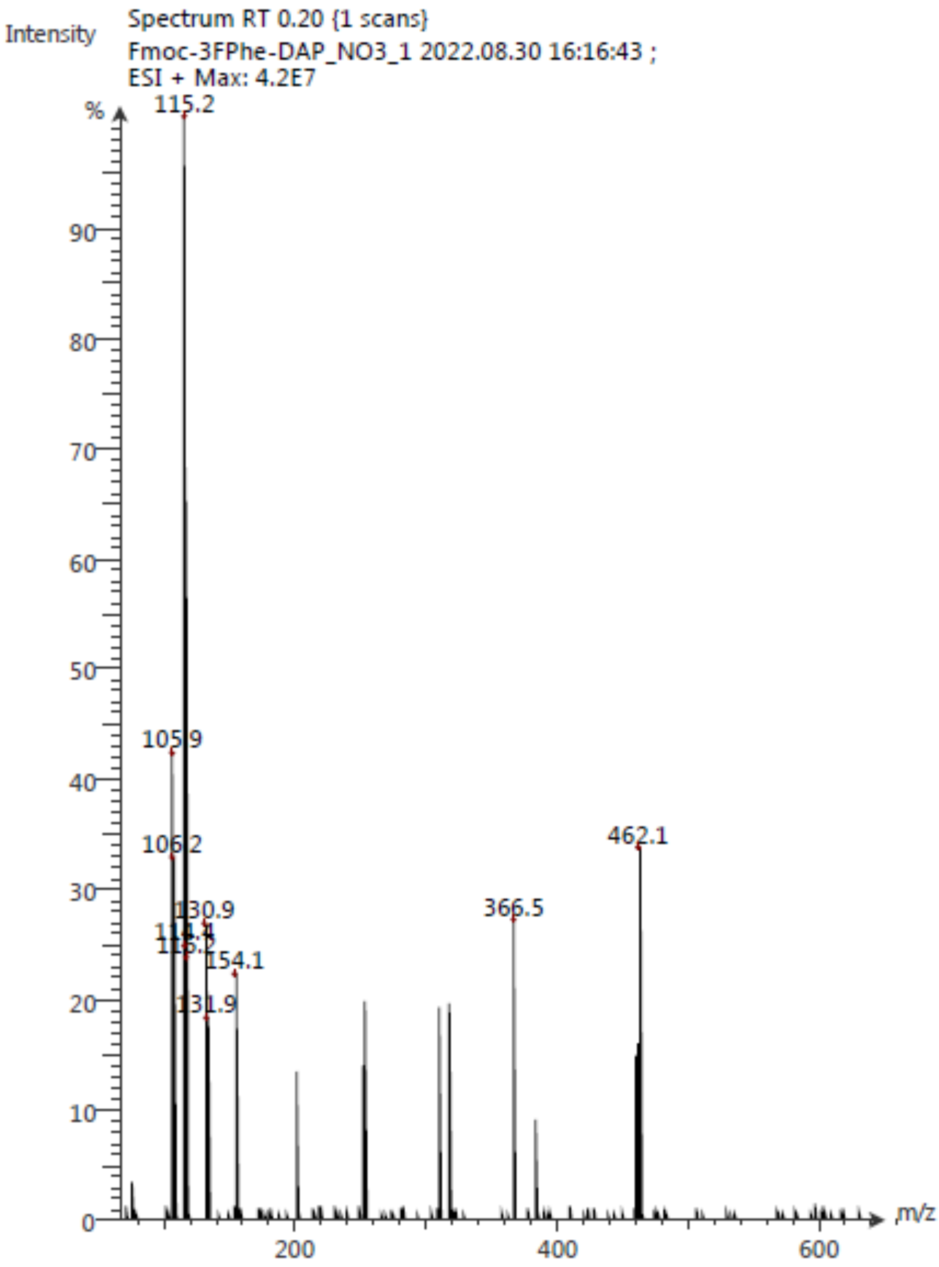

Figure S19. Mass spectrum of Fmoc-3F-Phe-DAP (**2**) after 1 week of gelation with 100 mM $NaNO_3$.

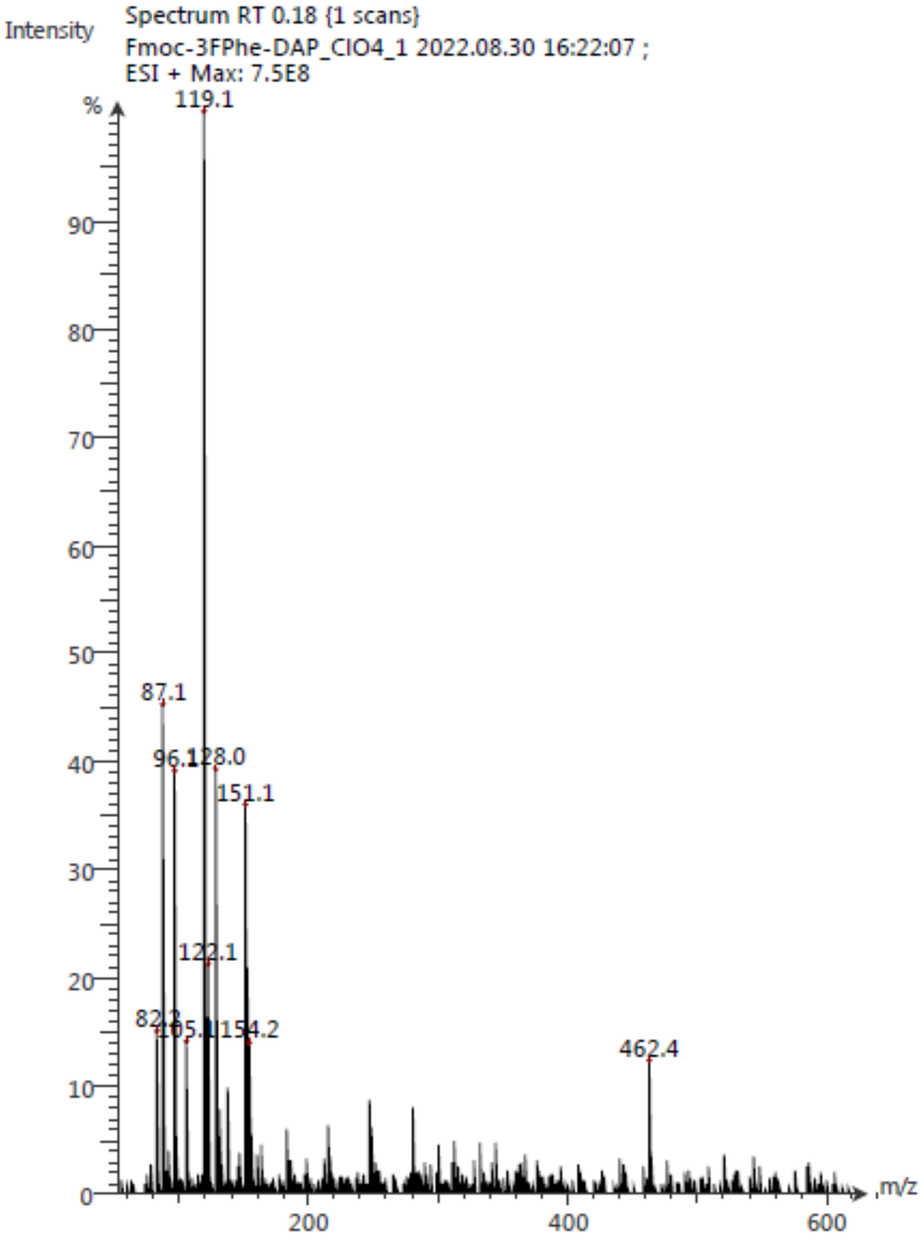

Figure S20. Mass spectrum of Fmoc-3F-Phe-DAP (**2**) after 1 week of gelation with 100 mM $NaClO_4$.

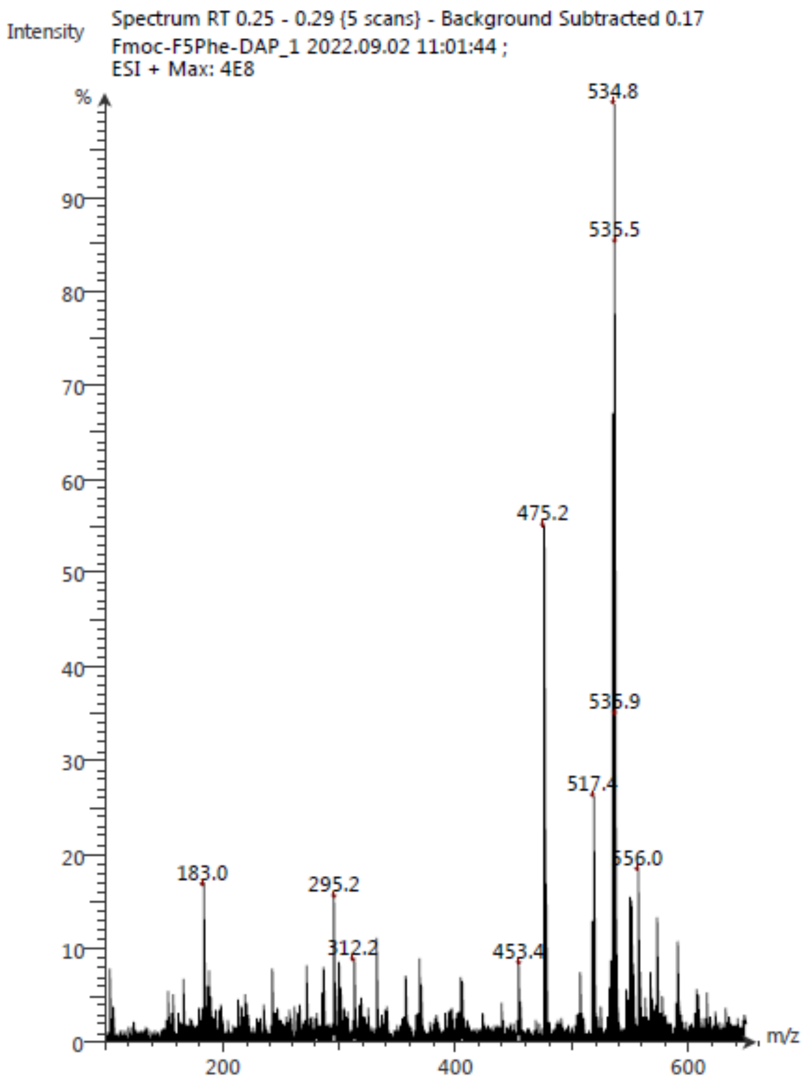

Figure S21. Mass spectrum of Fmoc-F_5-Phe-DAP (**3**).

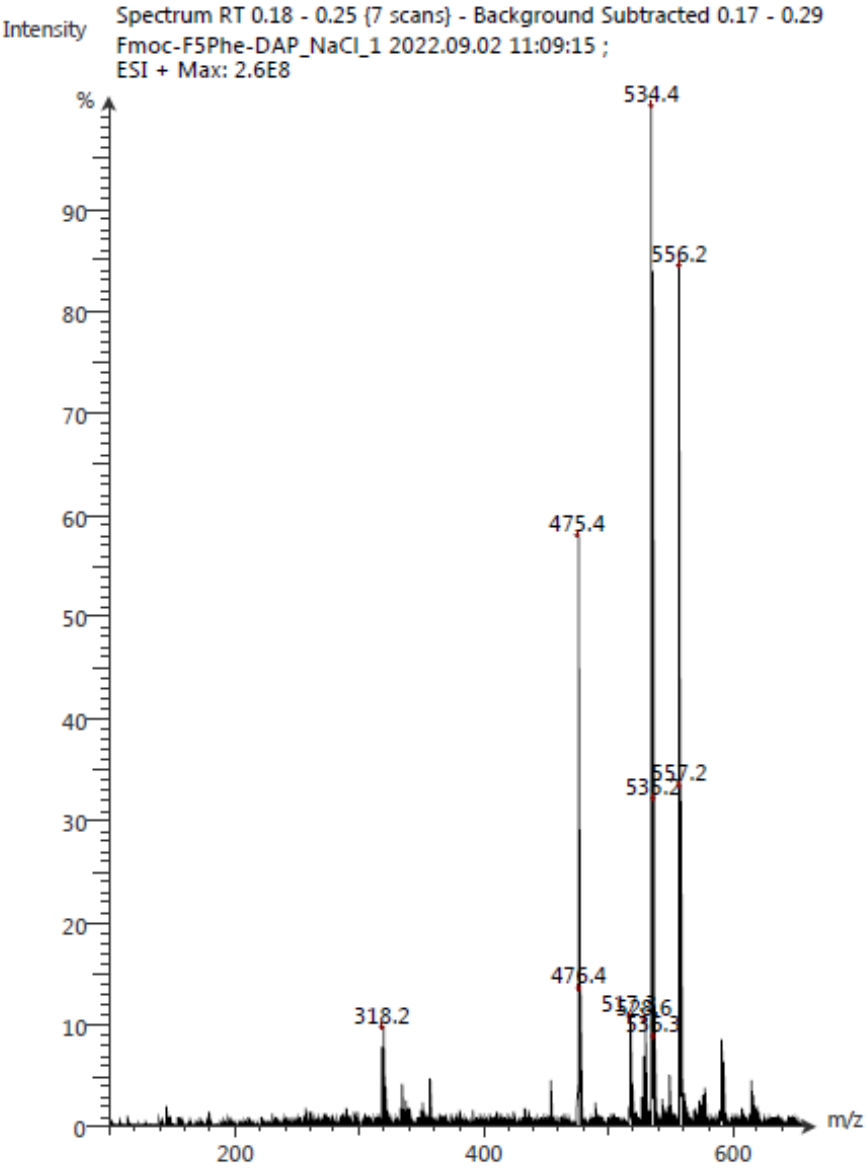

Figure S22. Mass spectrum of Fmoc-F_5-Phe-DAP (**3**) after 1 week of gelation with 100 mM NaCl.

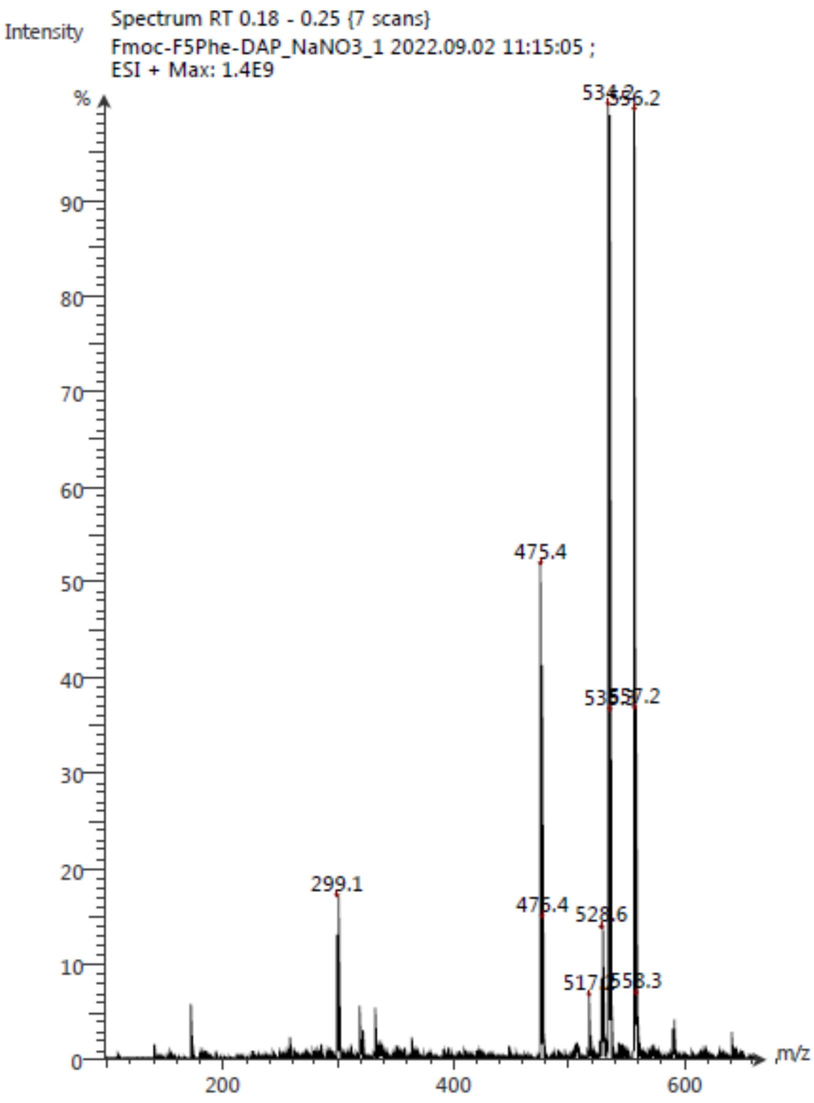

Figure S23. Mass spectrum of Fmoc-F_5-Phe-DAP (**3**) after 1 week of gelation with 100 mM $NaNO_3$.

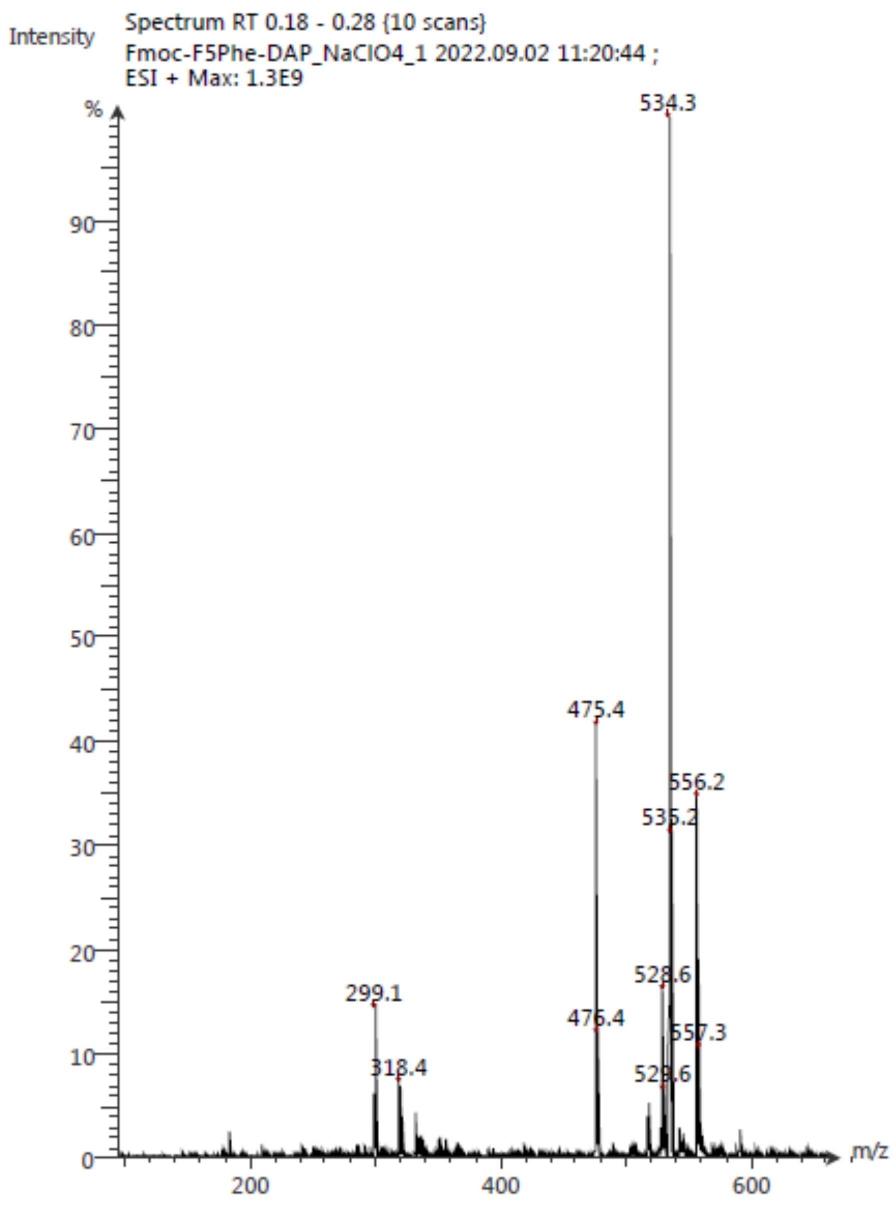

Figure S24. Mass spectrum of Fmoc-F_5-Phe-DAP (**3**) after 1 week of gelation with 100 mM $NaClO_4$.

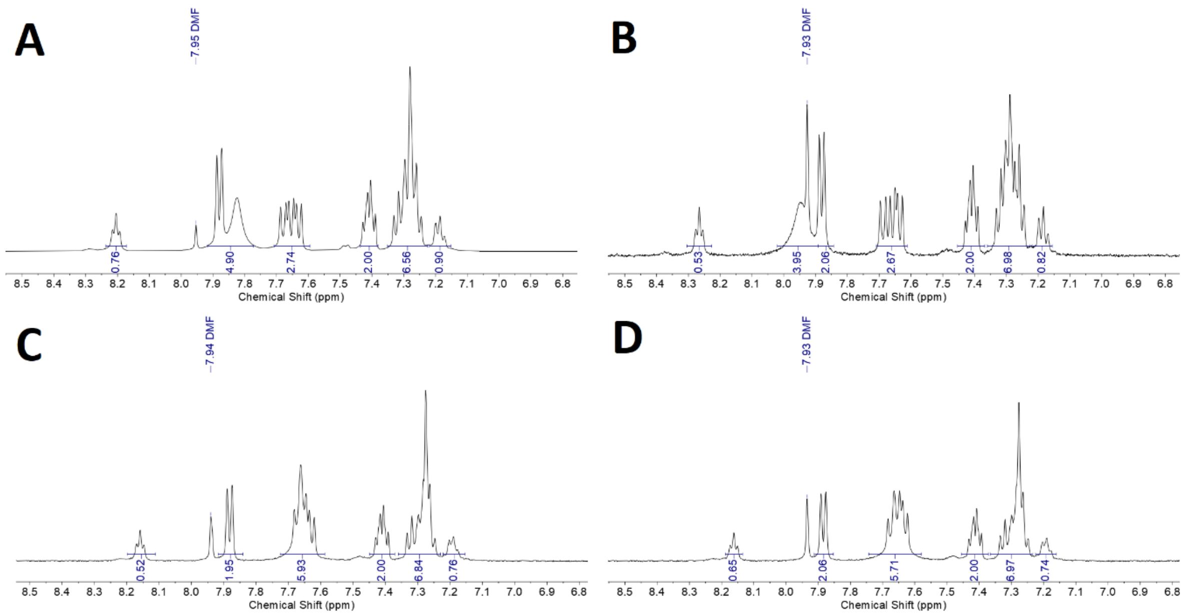

Figure S25. ^{1}H NMR spectra of Fmoc-Phe-DAP (**1**) in (A) monomeric form (DMSO-d$_6$), (B) after 1 week of gelation with 100 mM NaCl (DMSO-d$_6$), (C) after 1 week of gelation with 100 mM $NaClO_4$ (DMSO-d$_6$), (D) after 1 week of gelation with 100 mM $NaNO_3$ (DMSO-d$_6$). External standard of 24 mM DMF in DMSO-d_6 was inserted in a sealed capillary tube.

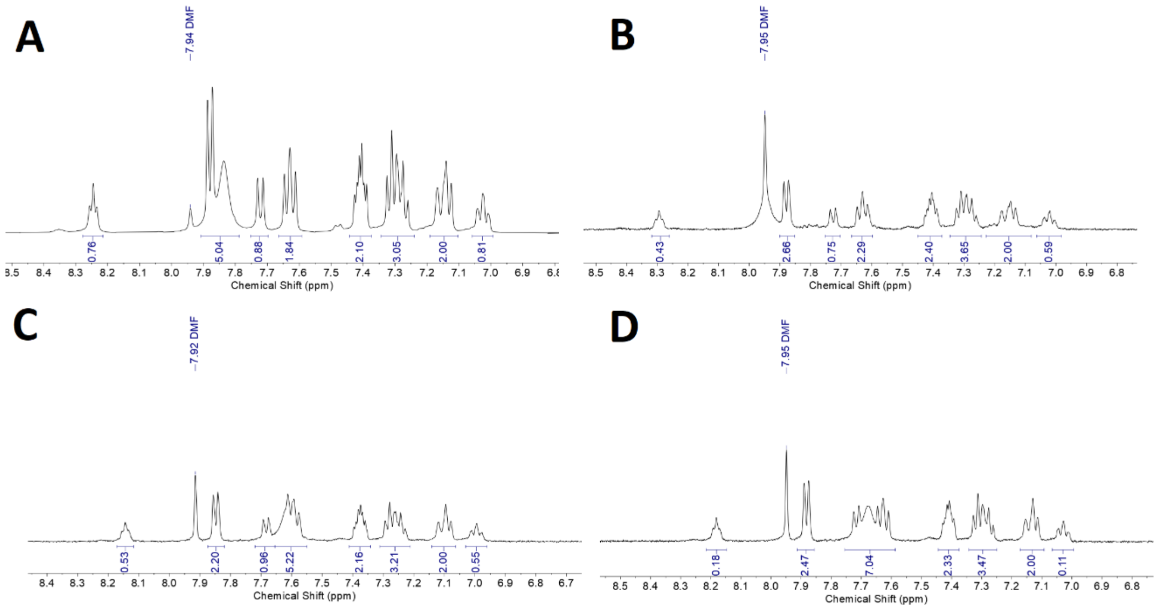

Figure S26. ^{1}H NMR spectra of Fmoc-3F-Phe-DAP (**2**) in (A) monomeric form (DMSO-d$_6$), (B) after 1 week of gelation with 100 mM NaCl (DMSO-d$_6$), (C) after 1 week of gelation with 100 mM $NaClO_4$ (DMSO-d$_6$), (D) after 1 week of gelation with 100 mM $NaNO_3$ (DMSO-d$_6$). External standard of 24 mM DMF in DMSO-d_6 was inserted in a sealed capillary tube.

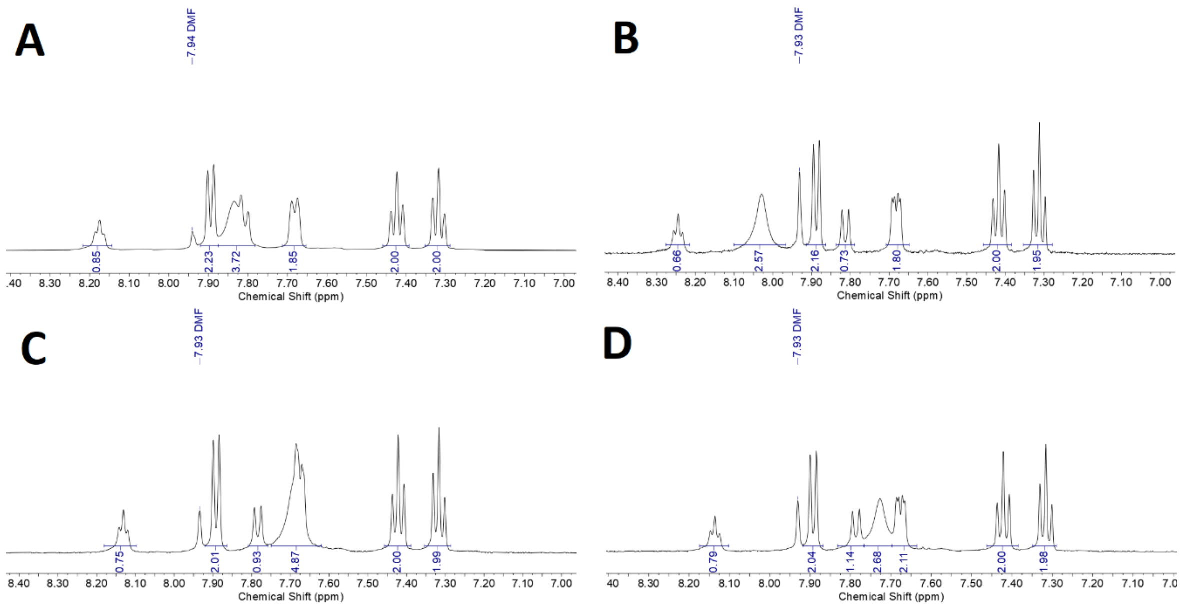

Figure S27. ^{1}H NMR spectra of Fmoc-F_5-Phe-DAP (**3**) in (A) monomeric form (DMSO-d_6), (B) after 1 week of gelation with 100 mM NaCl (DMSO-d_6), (C) after 1 week of gelation with 100 mM $NaClO_4$ (DMSO-d_6), (D) after 1 week of gelation with 100 mM $NaNO_3$ (DMSO-d_6). External standard of 24 mM DMF in DMSO-d_6 was inserted in a sealed capillary tube.

Table S4. Analytical HPLC method conditions. Each compound was injected onto an analytical HPLC instrument (Shimadzu 2010A) equipped with a Phenomenex Gemini 5 mmC18 column (250 × 4.6mm). A gradient of water (component A) and acetonitrile (component B), both containing 0.05% trifluoroacetic acid, was used as the mobile phase at a flow rate of 1 mL min^{-1} and UV detection was monitored at 215 nm. When assembled samples were analyzed, the assemblies were frozen, lyophilized, and resuspended in 1:1 acetonitrile:water solvent for analysis.

Method Time (min)	%B
0	5
5	5
15	95
20	95
22	5
25	5

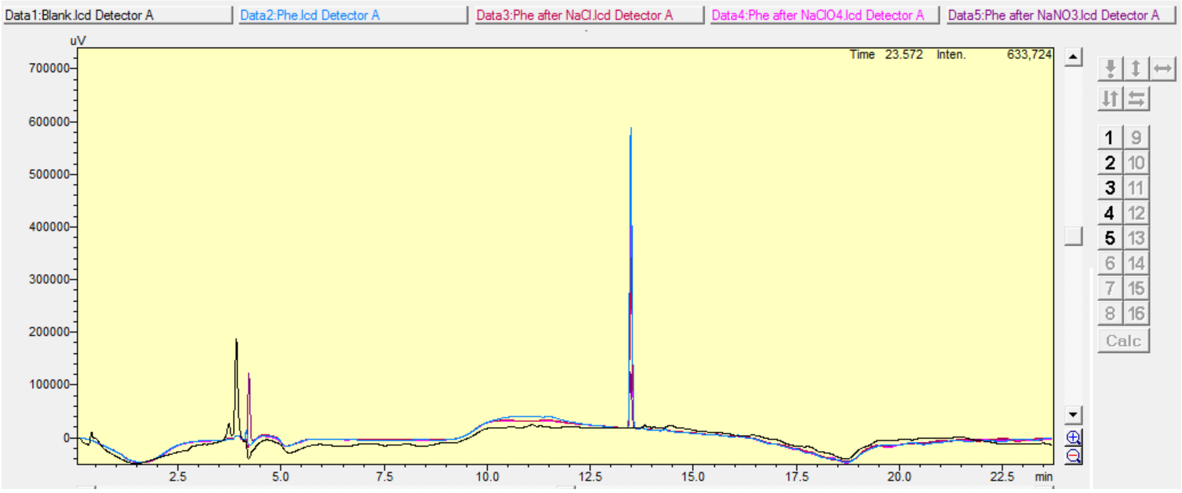

Figure S28. Analytical HPLC traces of Fmoc-Phe-DAP (**1**). Blue line: monomeric form; red line: after 1 week of gelation with 100 mM NaCl, pink line: after 1 week of gelation with 100 mM $NaClO_4$, purple line: after 1 week of gelation with 100 mM $NaNO_3$.

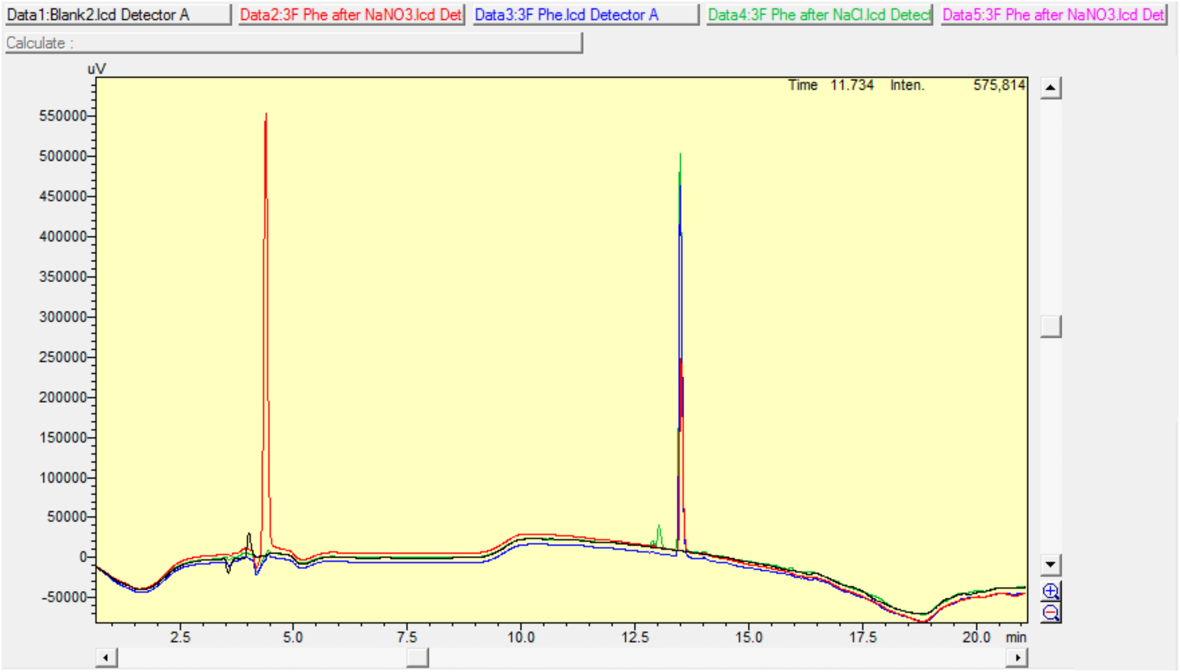

Figure S29. Analytical HPLC traces of Fmoc-3F-Phe-DAP (**2**). Blue line: monomeric form, green line: after 1 week of gelation with 100 mM NaCl, red line: after 1 week of gelation with 100 mM $NaClO_4$, pink line: after 1 week of gelation with 100 mM $NaNO_3$.

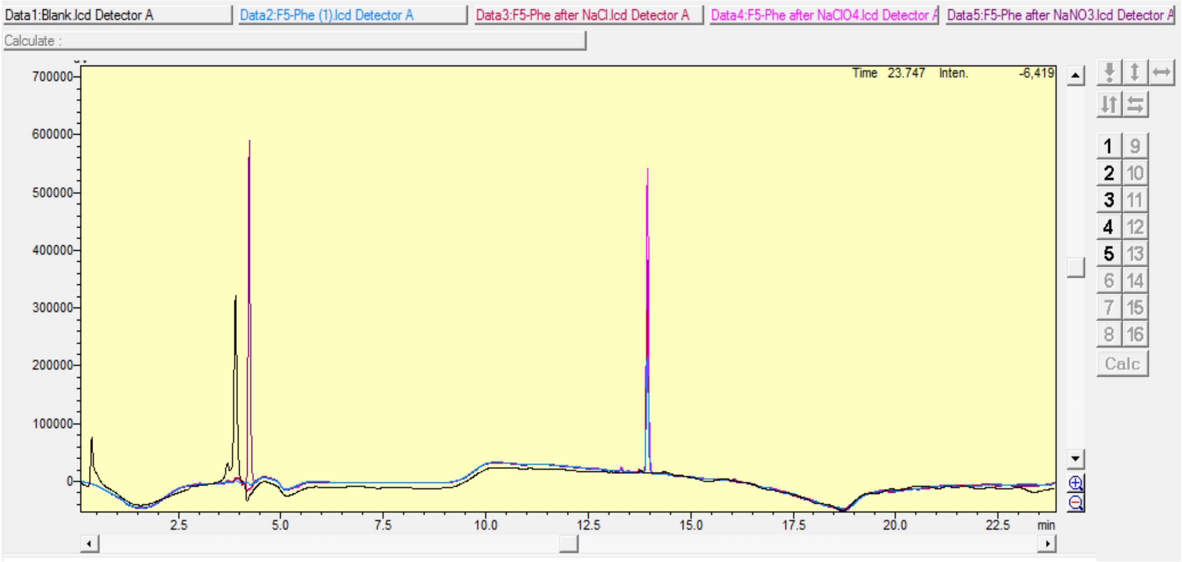

Figure S30. Analytical HPLC traces of Fmoc-F_5-Phe-DAP (**3**). Blue line: monomeric form, red line: after 1 week of gelation with 100 mM NaCl, pink line: after 1 week of gelation with 100 mM $NaClO_4$, purple line: after 1 week of gelation with 100 mM $NaNO_3$.

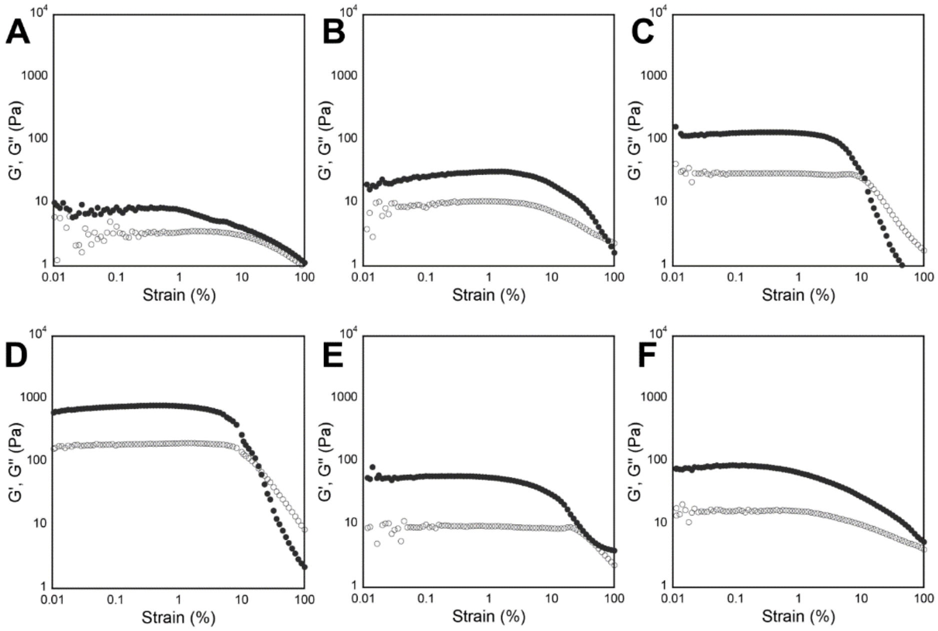

Figure S31. Strain sweep data collected via oscillatory rheology of 10 mM hydrogels of Fmoc-Phe-DAP (**1**) 24 hours after assembly. G′ and G″ values (Pa) are represented by closed circles and open circles, respectively. Hydrogels were formed by mixing with (A) NaOAc (B) NaCl (C) NaBr (D) $NaNO_3$ (E) NaI (F) $NaClO_4$.

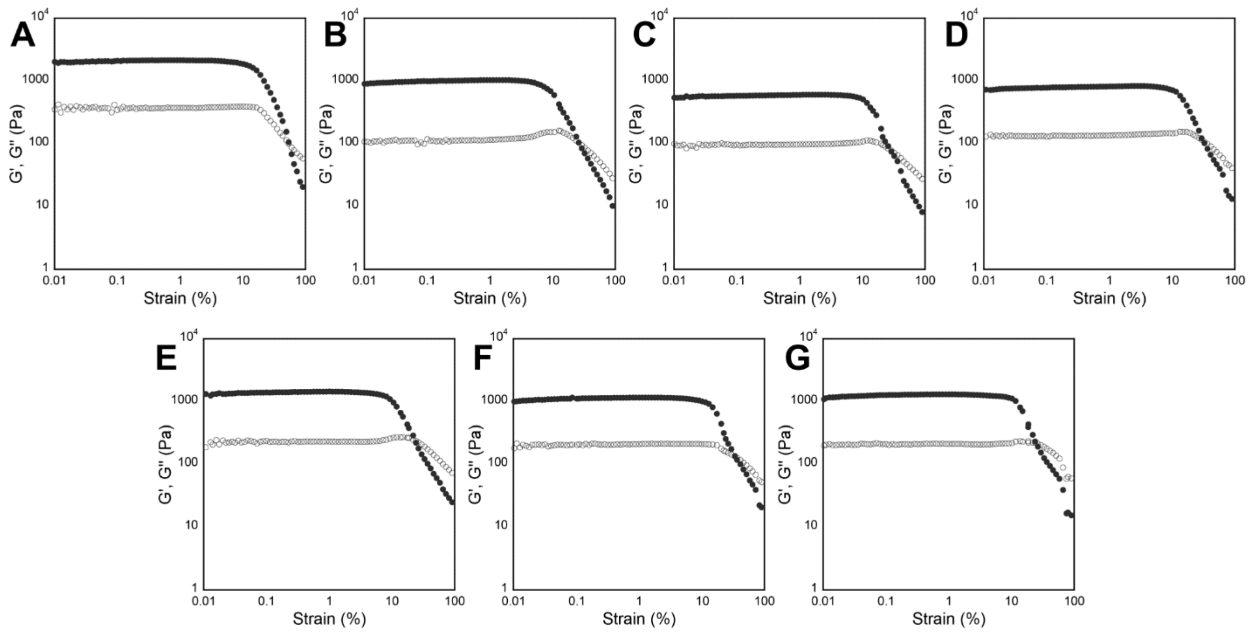

Figure S32. Strain sweep data collected via oscillatory rheology of 10 mM hydrogels of Fmoc-3F-Phe-DAP (**2**) 24 hours after assembly. G′ and G″ values (Pa) are represented by closed circles and open circles, respectively. Hydrogels were formed by mixing with (A) $Na_3C_6H_5O_7$ (B) NaOAc (C) NaCl (D) NaBr (E) $NaNO_3$ (F) NaI (G) NaSCN.

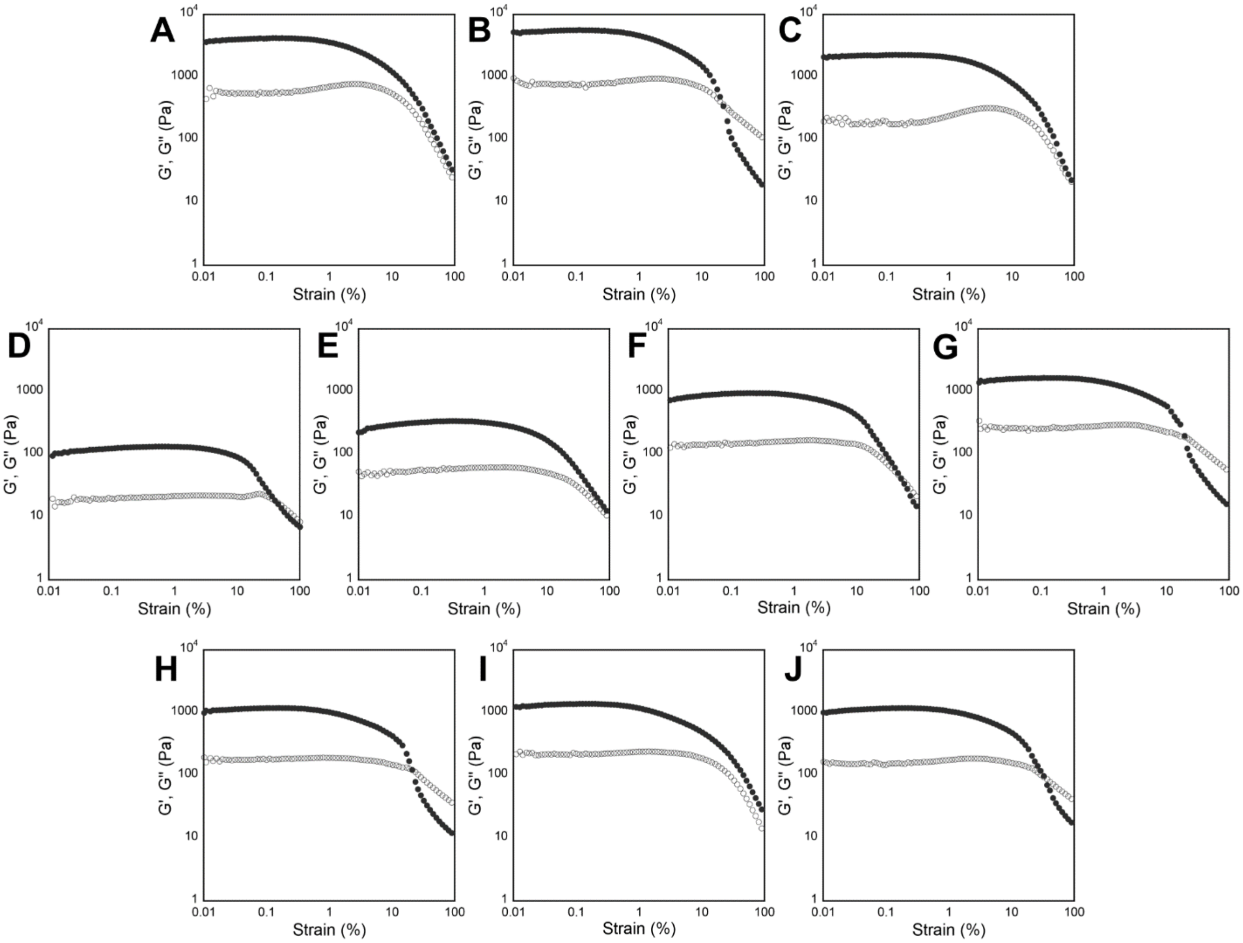

Figure S33. Strain sweep data collected via oscillatory rheology of 10 mM hydrogels of Fmoc-F_5-Phe-DAP (**3**) 24 hours after assembly. G′ and G″ values (Pa) are represented by closed circles and open circles, respectively. Hydrogels were formed by mixing with (A) $Na_3C_6H_5O_7$ (B) Na_2SO_4 (C) Na_2HPO_4 (D) NaOAc (E) NaCl (F) NaBr (G) $NaNO_3$ (H) NaI (I) $NaClO_4$ (J) NaSCN.

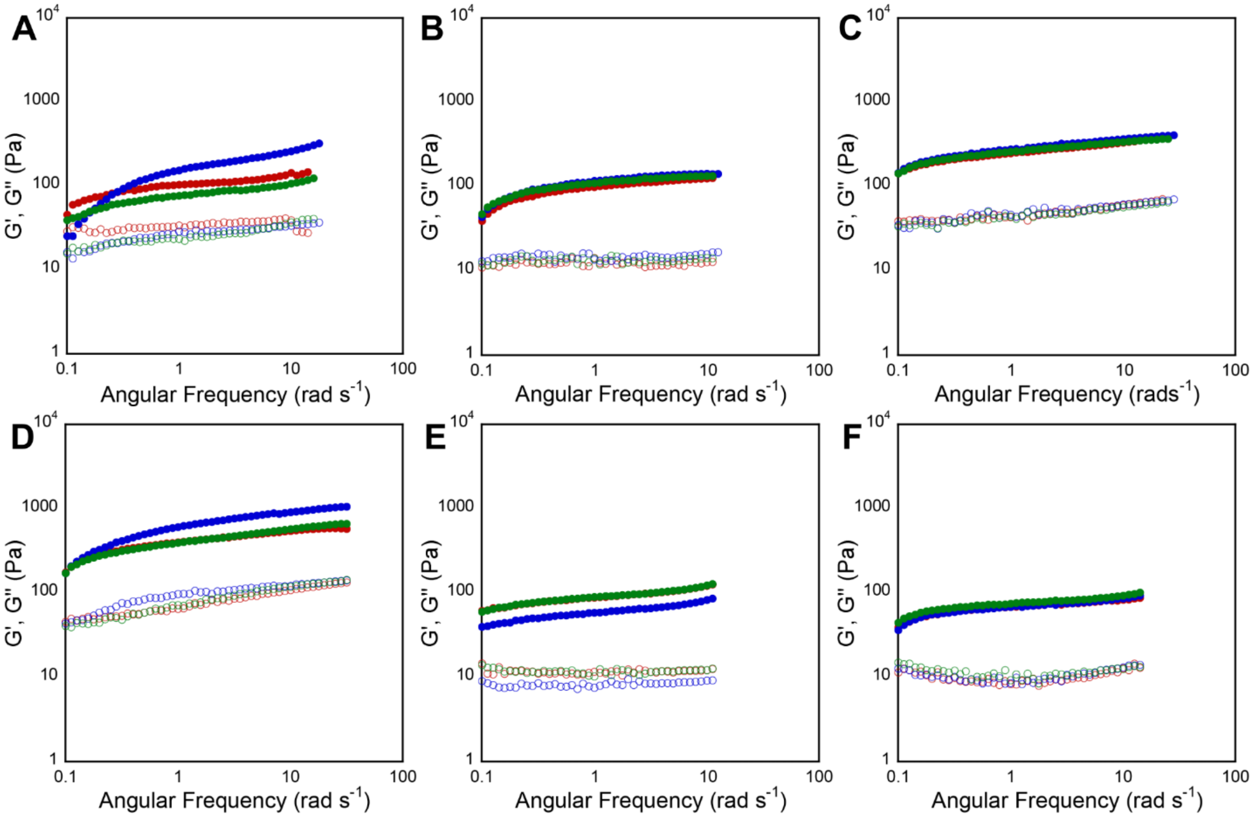

Figure S34. Frequency sweep data collected via oscillatory rheology of 10 mM hydrogels of Fmoc-Phe-DAP (**1**) 24 hours after assembly. G′ and G″ values (Pa) are represented by closed circles and open circles, respectively. Each plot contains three distinct measurements. Values at the upper end of the frequency sweep were cut off when the raw phase angle increased above 175º as recommended for the TA DHR series of rheometers, since values beyond this point are dominated by the instrument inertial torque instead of the sample torque.[1] Hydrogels were formed by mixing with (A) NaOAc (B) NaCl (C) NaBr (D) $NaNO_3$ (E) NaI (F) $NaClO_4$.

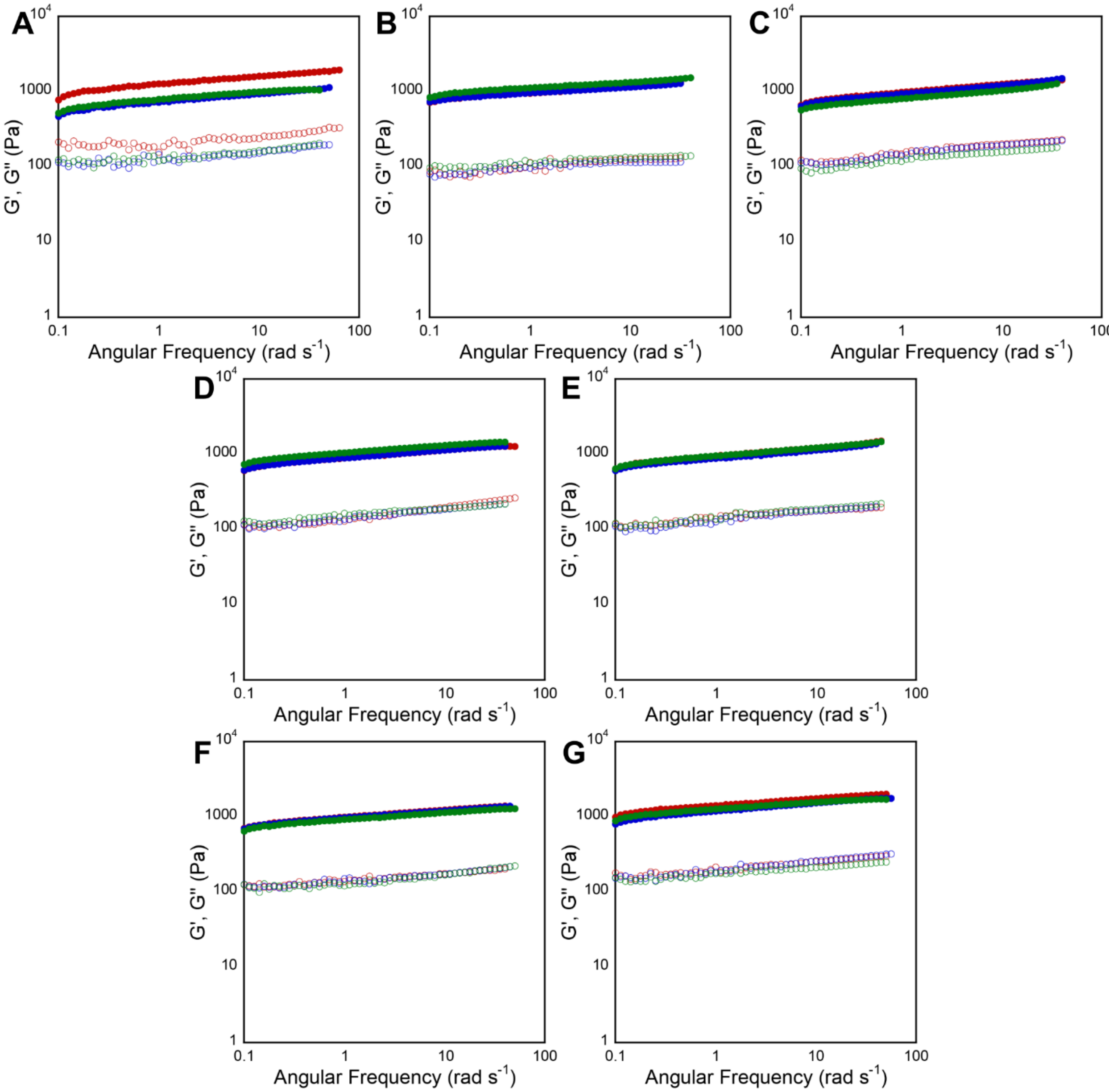

Figure S35. Frequency sweep data collected via oscillatory rheology of 10 mM hydrogels of Fmoc-3F-Phe-DAP (**2**) 24 hours after assembly. G′ and G″ values (Pa) are represented by closed circles and open circles, respectively. Each plot contains three distinct measurements. Values at the upper end of the frequency sweep were cut off when the raw phase angle increased above 175º as recommended for the TA DHR series of rheometers, since values beyond this point are dominated by the instrument inertial torque instead of the sample torque.[1] Hydrogels were formed by mixing with (A) $Na_3C_6H_5O_7$ (B) NaOAc (C) NaCl (D) NaBr (E) $NaNO_3$ (F) NaI (G) NaSCN.

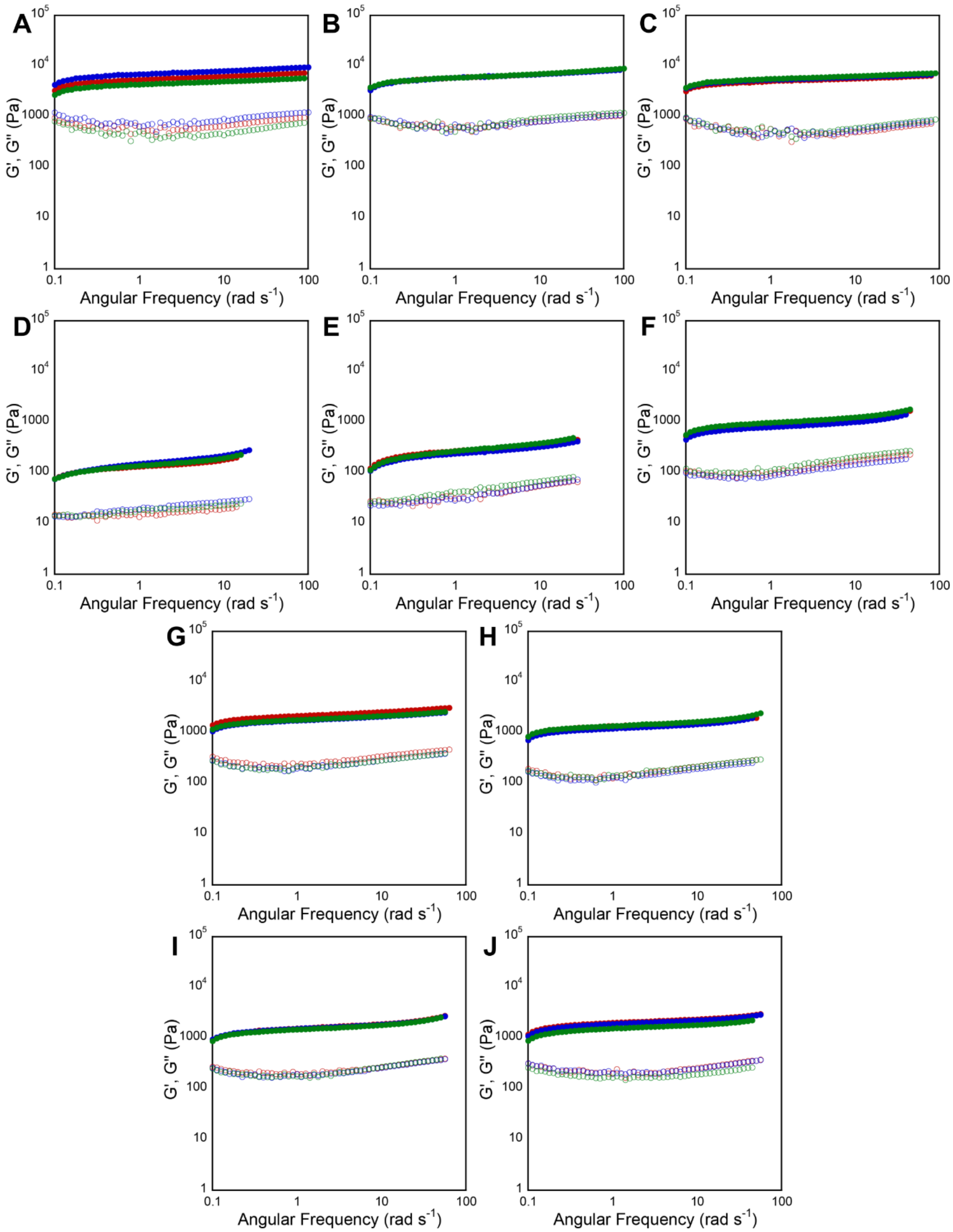

Figure S36. Frequency sweep data collected via oscillatory rheology of 10 mM hydrogels of Fmoc-F_5-Phe-DAP (**3**) 24 hours after assembly. G′ and G″ values (Pa) are represented by closed circles and open circles, respectively. Each plot contains three distinct measurements. Values at the upper end of the frequency sweep were cut off when the raw phase angle increased above 175° as recommended for the TA DHR series of rheometers, since values beyond this point are dominated by the instrument inertial torque instead of the sample torque.[1] Hydrogels were formed by mixing with (A) $Na_3C_6H_5O_7$ (B) Na_2SO_4 (C) Na_2HPO_4 (D) NaOAc (E) NaCl (F) NaBr (G) $NaNO_3$ (H) NaI (I) $NaClO_4$ (J) NaSCN.

Table S5. Average G′ and G″ for each hydrogel calculated from frequency sweeps shown in **Figures S34–S36**. Data is in Pa units. Error is reported as the standard deviation about the mean.

	**Gelator**		
**Salt**	**Fmoc-Phe-DAP (1)**	**Fmoc-3F-Phe-DAP (2)**	**Fmoc-F_5-Phe-DAP (3)**
**$Na_3C_6H_5O_7$**	N/A	G′: 1022 ± 333<br>G″: 172 ± 52	G′: 5953 ± 1429<br>G″: 704 ± 181
**Na_2SO_4**	N/A	N/A	G′: 6446 ± 86<br>G″: 806 ± 36
**Na_2HPO_4**	N/A	N/A	G′: 5624 ± 317<br>G″: 591 ± 34
**NaOAc**	G′: 115 ± 43<br>G″: 29 ± 4	G′: 1077 ± 103<br>G″: 110 ± 9	G′: 148 ± 13<br>G″: 19 ± 2
**NaCl**	G′: 105 ± 8<br>G″: 14 ± 1	G′: 999 ± 100<br>G″: 155 ± 18	G′: 281 ± 19<br>G″: 43 ± 5
**NaBr**	G′: 279 ± 15<br>G″: 49 ± 2	G′: 1031 ± 91<br>G″: 163 ± 6	G′: 996 ± 109<br>G″: 137 ± 21
**$NaNO_3$**	G′: 515 ± 136<br>G″: 89 ± 9	G′: 1027 ± 48<br>G″: 154 ± 7	G′: 2054 ± 249<br>G″: 274 ± 30
**NaI**	G′: 80 ± 18<br>G″: 11 ± 2	G′: 1047 ± 37<br>G″: 153 ± 3	G′: 1431 ± 103<br>G″: 179 ± 12
**$NaClO_4$**	G′: 71 ± 4<br>G″: 11 ± 1	N/A	G′: 1686 ± 47<br>G″: 242 ± 9
**NaSCN**	N/A	G′: 1421 ± 114<br>G″: 212 ± 18	G′: 1943 ± 271<br>G″: 234 ± 33

Table S6. pH values of assemblies of **1–3** at 30 minutes, 24 hours, and 1 week after assembly.

		Gelator		
**Salt**	**Time**	**Fmoc-Phe-DAP (1)**	**Fmoc-3F-Phe-DAP (2)**	**Fmoc-F_5-Phe-DAP (3)**
**$Na_3C_6H_5O_7$**	30 min	8.5	8.1	7.8
	24 h	8.4	7.8	7.9
	1 week	8.3	7.6	8.1
**Na_2SO_4**	30 min	8.0	7.5	6.5
	24 h	8.2	7.3	7.0
	1 week	8.2	7.3	6.7
**Na_2HPO_4**	30 min	9.2	9.0	8.6
	24 h	9.1	8.7	8.3
	1 week	9.1	8.4	8.8
**NaOAc**	30 min	7.0	7.2	7.0
	24 h	7.0	7.1	7.1
	1 week	7.0	7.1	6.8
**NaCl**	30 min	6.4	7.1	6.5
	24 h	6.6	7.1	6.7
	1 week	6.8	7.0	6.3
**NaBr**	30 min	6.3	7.1	6.3
	24 h	6.6	7.1	6.5
	1 week	6.8	7.1	6.2
**$NaNO_3$**	30 min	6.8	7.2	6.2
	24 h	6.9	7.1	6.7
	1 week	7.0	7.2	6.4
**NaI**	30 min	6.2	7.1	5.9
	24 h	6.4	7.1	6.1
	1 week	6.8	7.1	6.0
**$NaClO_4$**	30 min	6.1	7.1	5.5
	24 h	6.3	7.2	5.8
	1 week	7.0	7.3	5.6
**NaSCN**	30 min	6.4	7.2	5.7
	24 h	6.6	7.2	6.0
	1 week	6.8	7.3	5.8

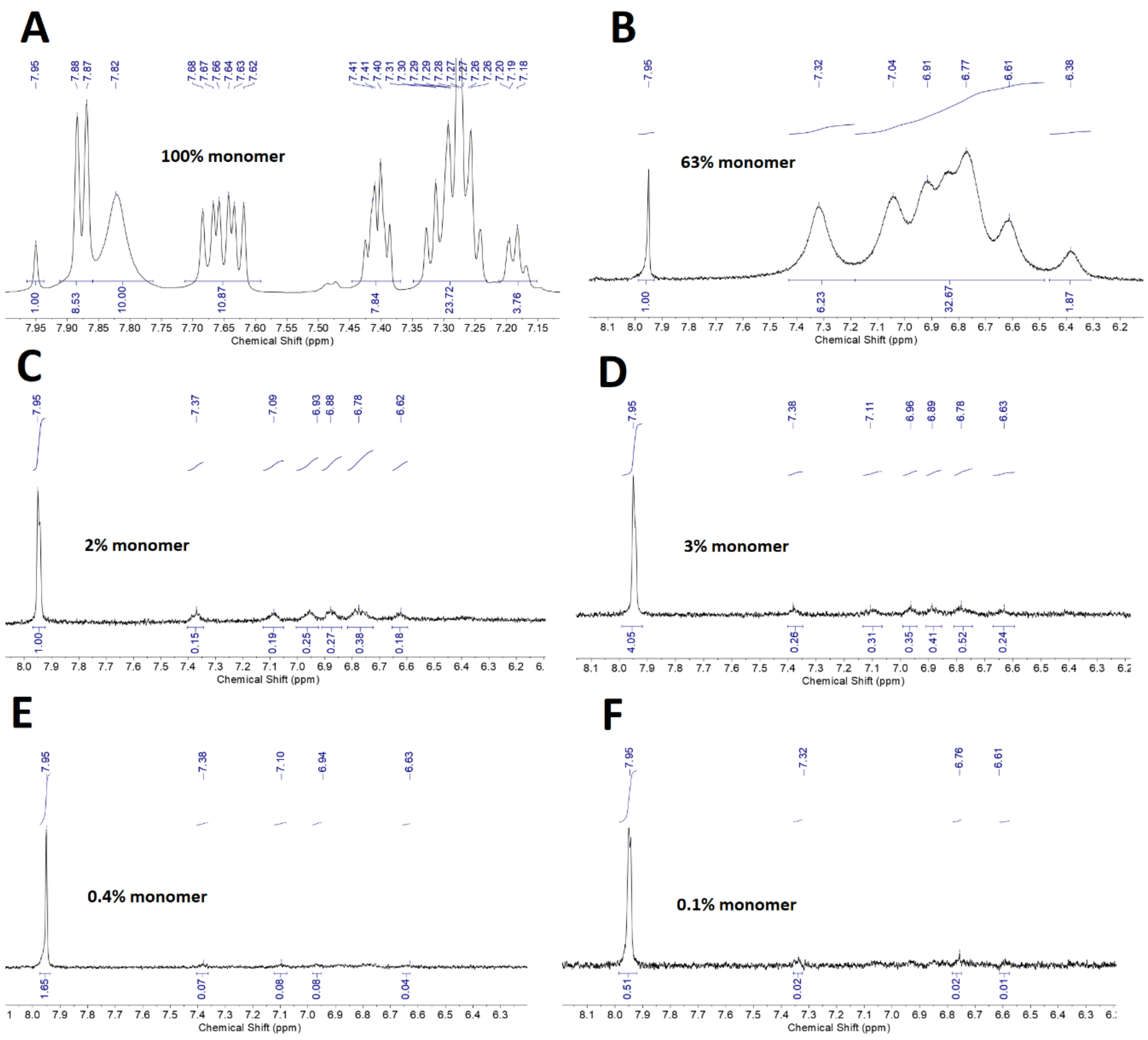

Figure S37. ^{1}H NMR spectra of Fmoc-Phe-DAP (**1**) at 10 mM. (A) monomeric form (DMSO-d_6), (B) solution form (D2O, no salt), (C) gel form (D_2O and 100 mM NaCl), (D) gel form (D_2O and 100 mM NaSCN), (E) gel form (D_2O and 100 mM $NaClO_4$), (F) gel form (D_2O and 100 mM $Na_3C_6H_5O_7$). Comparative integration to quantify monomer concentration was performed against an external standard of 24 mM DMF in DMSO-d_6 inserted in a sealed capillary tube.

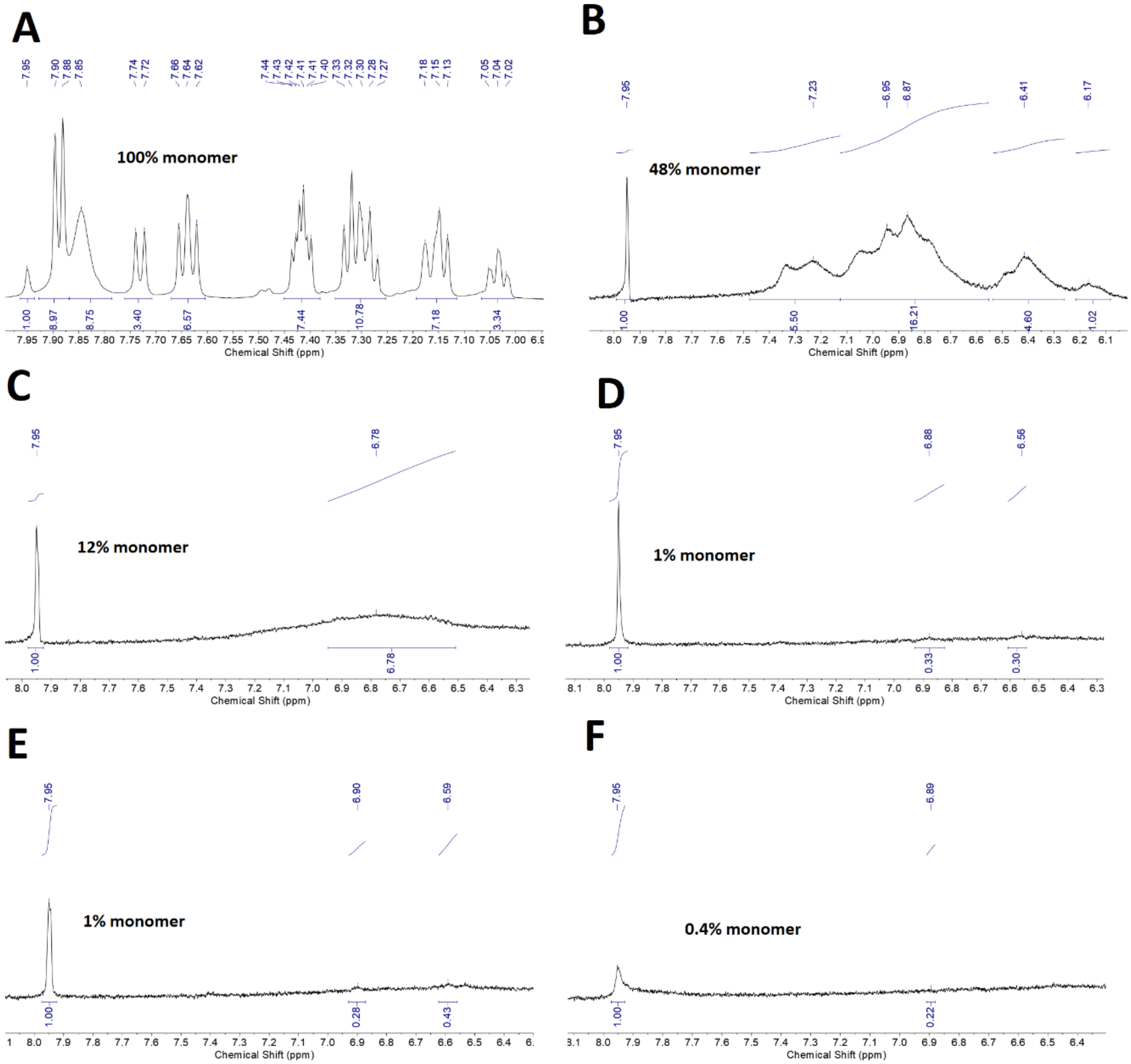

Figure S38. ^{1}H NMR spectra of Fmoc-3F-Phe-DAP (**2**) at 10 mM. (A) monomeric form (DMSO-d_6), (B) solution form (D_2O, no salt), (C) gel form (D_2O and 100 mM NaCl), (D) gel form (D_2O and 100 mM NaSCN), (E) gel form (D_2O and 100 mM $NaClO_4$), (F) gel form (D_2O and 100 mM $Na_3C_6H_5O_7$). Comparative integration to quantify monomer concentration was performed against an external standard of 24 mM DMF in DMSO-d_6 inserted in a sealed capillary tube.

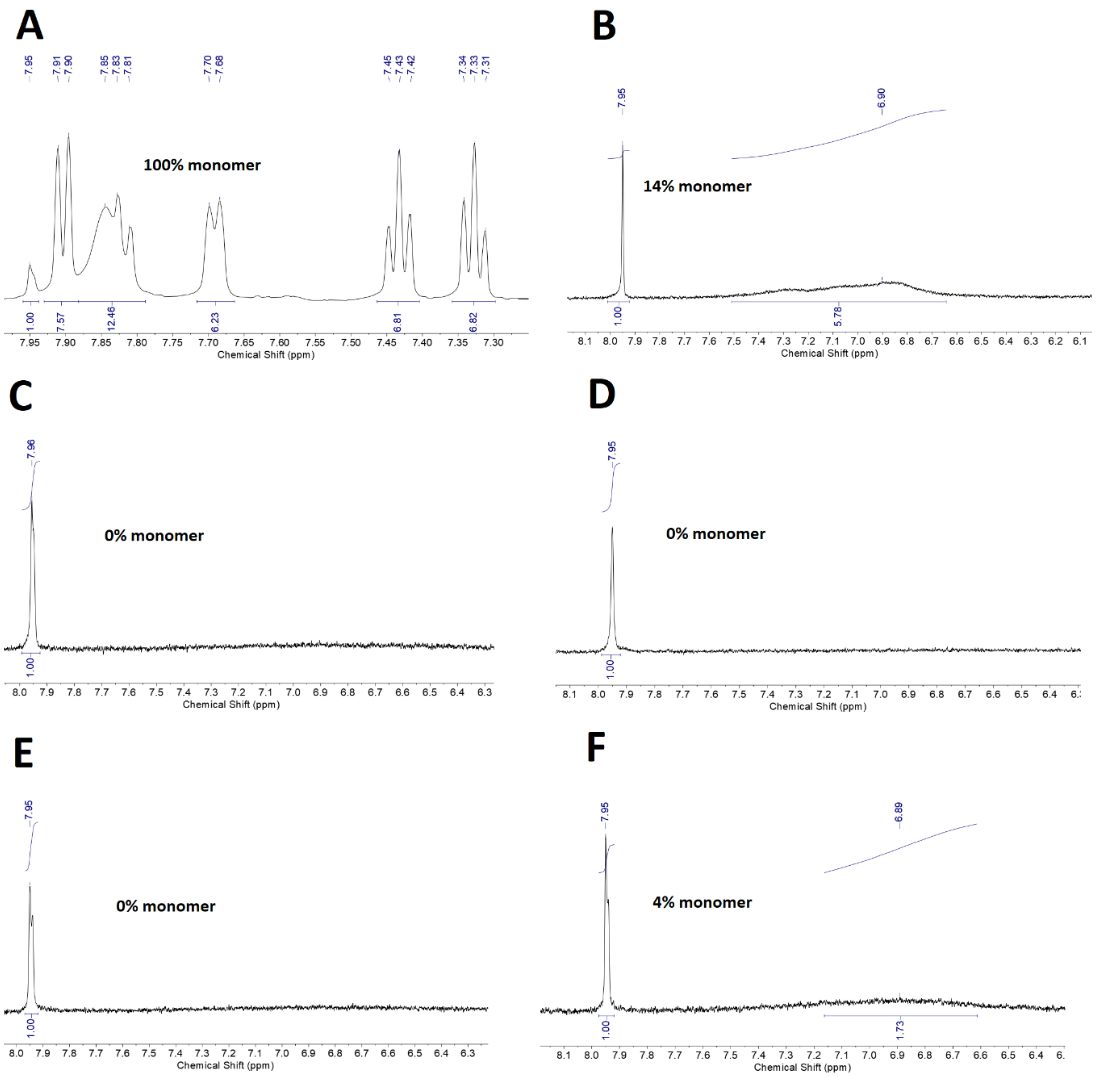

Figure S39. ^{1}H NMR spectra of Fmoc-F_5-Phe-DAP (**3**) at 10 mM. (A) monomeric form (DMSO-d_6), (B) solution form (D_2O, no salt), (C) gel form (D_2O and 100 mM NaCl), (D) gel form (D_2O and 100 mM NaSCN), (E) gel form (D_2O and 100 mM $NaClO_4$), (F) gel form (D_2O and 100 mM $Na_3C_6H_5O_7$). Comparative integration to quantify monomer concentration was performed against an external standard of 24 mM DMF in DMSO-d_6 inserted in a sealed capillary tube.

References

1. Ewoldt, R. H.; Johnston, M. T.; Caretta, L. M., Experimental Challenges of Shear Rheology: How to Avoid Bad Data. In *Complex Fluids in Biological Systems: Experiment, Theory, and Computation*, Spagnolie, S. E., Ed. Springer: New York, NY, 2015; pp 207-241.
